# Gene therapy enhances deoxyribonuclease I treatment in antimyeloperoxidase glomerulonephritis

**DOI:** 10.1172/jci.insight.188951

**Published:** 2025-07-08

**Authors:** Anne Cao Le, Virginie Oudin, Jonathan Dick, Maliha A. Alikhan, Timothy A. Gottschalk, Lu Lu, Kate E. Lawlor, Daniel Koo Yuk Cheong, Mawj Mandwie, Ian E. Alexander, A.R. Kitching, Poh-Yi Gan, Grant J. Logan, Kim M. O’Sullivan

**Affiliations:** 1 Centre for Inflammatory Diseases, Monash University Department of Medicine, Clayton, Victoria, Australia.; 2 Centre for Innate Immunity and Infectious Diseases, Hudson Institute of Medical Research, Clayton, Victoria, Australia.; 3 Department of Molecular and Translational Science, Monash University, Clayton, Victoria, Australia.; 4 Gene Therapy Research Unit, Children’s Medical Research Institute and Sydney Children’s Hospitals Network, University of Sydney, New South Wales, Sydney, Australia.; 5 Discipline of Child and Adolescent Health, University of Sydney, Sydney, New South Wales, Australia.; 6 Departments of Nephrology and Pediatric Nephrology, Monash Health, Melbourne, Victoria, Australia.

**Keywords:** Autoimmunity, Inflammation, Autoimmune diseases, Neutrophils, Vasculitis

## Abstract

Extracellular DNA (ecDNA) released from injured and dying cells powerfully induces injurious inflammation. In this study we define the role of ecDNA in systemic vasculitis affecting the kidney, using human kidney biopsies and murine models of myeloperoxidase anti-neutrophil cytoplasmic antibody-associated glomerulonephritis (MPO-ANCA GN). Twice daily administration of intravenous deoxyribonuclease I (ivDNase I) in 2 models of anti-MPO GN reduced glomerular deposition of ecDNA, histological injury, leukocyte infiltration, and NETosis. Comprehensive investigation into DNase I modes of action revealed that after exposure to MPO, DNase I reduced lymph node DC numbers and their activation status, resulting in decreased frequency of MPO-specific CD4^+^ effector T cells (IFN-γ and IL-17A producing) and reductions in dermal anti-MPO delayed type hypersensitivity responses. To overcome the translational obstacle of the short half-life of DNase I (<5 hours), we tested an adeno-associated viral vector encoding DNase I. This method of DNase I delivery was more effective, as in addition to the histological and antiinflammatory changes described above, a single vector treatment also reduced circulating MPO-ANCA titers and albuminuria. These results indicate ecDNA is a potent driver of anti-MPO GN and DNase I is a potential therapeutic that can be delivered using gene technology.

## Abstract 2

Targeting proinflammatory extracellular DNA with DNase I to reduce inflammation and the deveopment of glomeruonephritis in MPO-ANCA Vasculitis.

## Introduction

Kidney involvement in anti-neutrophil cytoplasmic antibody–associated (ANCA-associated) vasculitis is common, often resulting in the development of glomerulonephritis (GN). If left untreated, disease will progress to end-stage renal disease requiring dialysis or a kidney transplant. ANCA-GN encompasses a group of autoimmune diseases characterized by inflammation of small- to medium-sized blood vessels, most commonly manifesting as granulomatosis with polyangiitis, microscopic polyangiitis, and eosinophilic granulomatosis with polyangiitis. These conditions are associated with autoantibodies targeting either proteinase 3 (PR3) or myeloperoxidase (MPO), leading to tissue-damaging neutrophil activation. PR3-ANCA and MPO-ANCA GN represent distinct disease subtypes with differing clinical features, genetic susceptibilities, and geographic distributions (1). PR3-ANCA disease is more prevalent in northern Europe and North America and is associated with HLA-DP variants whereas MPO-ANCA GN is more common in East Asia and Australia and is associated with HLA-DQ variants. In this study, we focus exclusively on MPO-ANCA vasculitis, as this is the predominant type in Australia, which we can model in a well-established murine model of anti-MPO GN (2). PR3-ANCA disease remains difficult to model because of the poor homology between human and murine PR3, limiting its study in vivo (1).

Current treatments for MPO-ANCA GN have reduced the number of deaths but have significant side effects, both in their detrimental effects on protective immunity and in their metabolic effects, leading to infections, malignancy, and cardiovascular disease (1, 3). These treatments, although relatively effective, target and ablate components of the immune system. Cyclophosphamide ablates immune cells from the bone marrow, and rituximab, a plasma cell–depleting antibody, prevents the production of antibody, rendering patients severely immunocompromised (1). Furthermore, corticosteroids used to dampen inflammation cause unwanted weight gain, brain fog, and interference with daily living. Understanding the underlying disease mechanisms of MPO-ANCA GN affords the opportunity to develop new therapeutics that target critical components of nephritogenic immune pathways and potentially offer safer, more specific treatments.

MPO-ANCA GN has a characteristic pattern of glomerular injury with considerable cell death and necrosis (4–7). Cellular injury or cell death can release pro-inflammatory damage-associated molecular patterns (DAMPs), including nuclear release of DNA as extracellular DNA (ecDNA). Neutrophils, which are the critical disease-initiating cells in MPO-ANCA GN, produce a unique form of cell death known as neutrophil extracellular traps (NETs), which expel large strands of DNA and are identified through decondensation of chromatin. This process relies heavily on reactive oxygen species (ROS) and protein-arginine deiminase 4–dependent (PAD4-dependent) citrullination of histones (8–12). NETs are thought to occur to increase the bactericidal killing capacity of neutrophils by extending the surface area in which to entrap bacteria (8, 13). However, in the context of sterile inflammation, NETs are highly injurious, releasing over 70 known pro-inflammatory mediators (14, 15). Fragments of ecDNA (including CpG oligonucleotides) are a major product of NETs, as well as other forms of cell death (apoptosis, necroptosis, necrosis), and drive inflammatory gene expression. Of particular interest is detection of ecDNA and signaling through cytosolic and membrane-bound DNA sensors, such as Toll-like receptor 9 (TLR9), stimulator of interferon genes, and cyclic GMP-AMP synthase, as they induce inflammatory responses in sterile inflammation through NF-κB nuclear translocation, type 1 interferon (IFN) cytokine production, and inflammasome activation (12, 16–23).

We have previously shown in models of sterile experimental anti-MPO GN and in MPO-ANCA GN patient kidney biopsies that TLR9 is upregulated, suggesting that DNA sensing is involved in the pathogenesis of MPO-ANCA GN. Furthermore, TLR9 mice are protected from the development of anti-MPO GN (24, 25). In host defense, excessive DNA release is controlled by endogenous deoxyribonuclease (DNase) enzymes. However, if cellular injury is severe, the production of ecDNA may exceed the capacity for DNase clearance thereby further driving inflammation. Loss of DNase I expression or functional exhaustion of DNase activity has been associated with heightened immune activation in other settings of autoimmune and inflammatory disease. For example, genetic deficiency or reduced DNase I activity has been linked to lupus-like disease, characterized by impaired clearance of NETs and enhanced stimulation of DNA-sensing pathways (26–28). Additionally, studies show that DNase I deficiency leads to NET accumulation, intravascular thrombosis, and systemic autoimmunity, highlighting the importance of maintaining ecDNA clearance (28, 29). This dysregulation can promote autoantibody formation, immune complex deposition, and sustained type I IFN production — features also observed in severe MPO-ANCA GN. These findings underscore the importance of DNase I in maintaining immunological tolerance and preventing unchecked inflammation and support the therapeutic rationale for DNase I supplementation in MPO-ANCA GN.

The kidney is a major source of DNase I outside the digestive system, and gene deletion of the enzyme in experimental mice induces GN (30–34). These observations support the possibility that DNase I removal of ecDNA is an important physiological process in the renal system. In support of this possibility, sera from patients with acute MPO-ANCA GN have a significant reduction of DNase I and higher levels of serum ecDNA when compared with healthy donors (35). NETs occur at high frequency within the glomeruli of patients with MPO-ANCA GN and are a large source of ecDNA (17, 20, 36). Importantly, it has been observed that MPO, the autoantigen in this disease, exerts enhanced biological effects when tethered to the ecDNA in NETs (37). These findings support the notion that ecDNA released from injured and dying glomerular cells and NETs can exceed the capacity for DNase I clearance, resulting in ecDNA accumulation in acute disease. The findings also support the possibility that exogenous DNase I administration may restore homeostatic clearance of ecDNA to relieve pathogenic inflammation.

In the current study, we sought to confirm whether ecDNA accumulation occurs in MPO-ANCA GN. Kidneys from patients with acute MPO-ANCA GN exhibited increased ecDNA, as well as reduced renal DNase I expression levels compared with control kidneys. The functional relevance of these phenomena was demonstrated in 2 murine models of anti-MPO GN, where disease is mediated by MPO-specific T cells or by anti-MPO antibodies. Importantly, intravenous administration of exogenous DNase I (ivDNase I) in 2 experimental models (cell mediated and MPO-ANCA mediated) markedly attenuated the development of GN, indicating that DNase I is a potential treatment for MPO-ANCA GN. To overcome the translational obstacle of the short half-life of DNase I in serum (<5 hours), we explored the use of DNase I gene transfer using an adeno-associated viral vector (AAV, vec-DNase I) to convert the liver into a biofactory for enzyme secretion into the circulatory system. The continuous supply of vec-DNase I in active murine anti-MPO GN showed enhanced therapeutic efficacy over twice daily doses of ivDNase I, demonstrated by better preservation of glomerular histology and reductions in MPO-ANCA and albuminuria. Collectively these findings support the pursuit of DNase I for the treatment of MPO-ANCA GN through DNase I gene therapy.

## Results

### EcDNA accumulates in glomeruli of patients with MPO-ANCA vasculitis.

To semiquantitate the extent of ecDNA accumulation in kidneys of patients with MPO-ANCA GN presenting with acute GN, we analyzed tissues collected from patients with this disease (*n* = 29) and compared them with control tissues collected from patients with a nonproliferative form of GN, minimal change disease (MCD; *n* = 6). Patients with MPO-ANCA GN all had a positive serum MPO-ANCA assay and presented with severe renal disease with an estimated glomerular filtration rate (eGFR) of 30 ± 8 (median ± 28.20 with a minimum of 10 and maximum of 105, mL/min/1.73^2^) compared with MCD that had the expected normal renal function (eGFR 100 ± 6). There were a mean of 18 glomeruli per biopsy (see other patient clinical data in Supplemental Table 1; supplemental material available online with this article; https://doi.org/10.1172/jci.insight.188951DS1).

**Figure 1:**
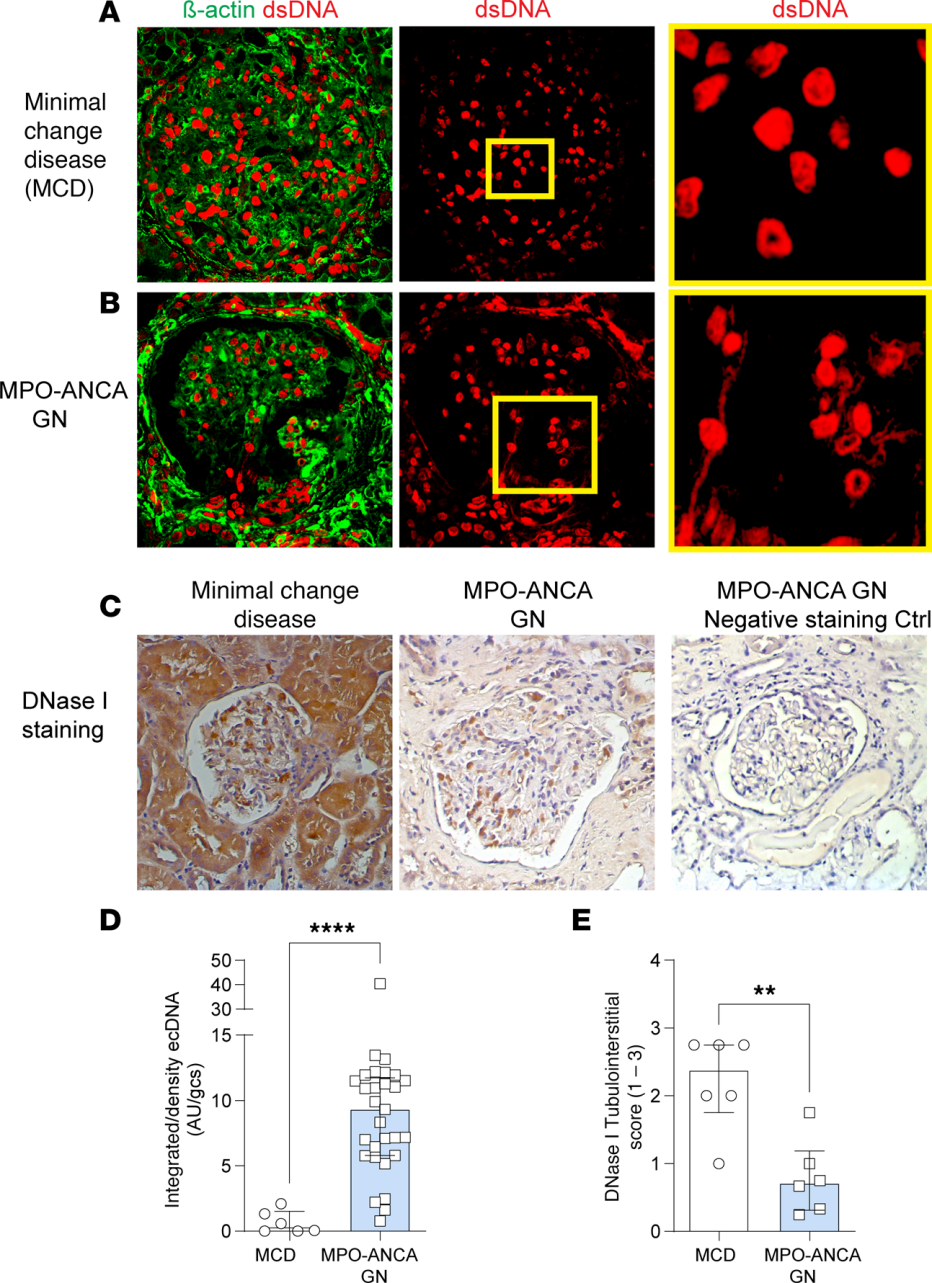
Enhanced ecDNA deposition and reduced DNase I expression in kidney biopsies from patients with MPO-ANCA GN. Renal biopsies from patients with ANCA vasculitis were stained for dsDNA (red) and counterstained with β-actin (green) to aid in identification of glomeruli and cells. (**A**) Biopsies from patients with minimal change disease (MCD) (with minimal glomerular injury) served as controls. Inset box shows higher power field of view with minimal ecDNA. (**B**) Patients with MPO-ANCA GN show extensive peri-glomerular and glomerular ecDNA. (**C**) MCD patients show extensive DNase I expression compared with the tubulointerstitium of patients with MPO-ANCA GN, and negative control for antibody specificity verifies the marked diminution of DNase I in MPO-ANCA GN. (**D**) Semiquantitation of extracellular dsDNA deposition in MPO-ANCA GN patients. (**E**) Semiquantification of DNase I in the tubulointerstitium of kidney biopsies. Score 1–3 indicates intensity of staining, with 0 indicating no staining, 1 minimal staining, 2 moderate staining, and 3 intense staining. **P < 0.005, ****P < 0.0001 Data are median (IQR). Human data are from *n* = 29 (MPO-ANCA GN) and *n* = 6 (MCD) in each group and analyzed by Mann-Whitney *U* test. Original magnification 400×, HP inset 3,000×. MPO, myeloperoxidase; IQR, interquartile range; ANCA anti-neutrophil cytoplasmic antibody; GN, glomerulonephritis; Ctrl, control; DNase I, deoxyribonuclease I; dsDNA, double-stranded deoxyribonucleic acid, ecDNA, extracellular deoxyribonucleic acid.

Renal biopsies were analyzed using a methodology that detects ecDNA regardless of the type of cell death (i.e., apoptotic, necrotic, or NETotic). Fragmented ecDNA outside the nucleus of cells was assessed by semiquantitative analysis of positive staining and expressed in arbitrary units (AU) per a previously validated method (38). There was significantly greater ecDNA deposition within the glomeruli of MPO-ANCA GN biopsies compared with control biopsies (Figure 1, A and B). It has been reported that patients with MPO-ANCA GN have lower levels of circulating DNase I (17), which may impair NET digestion and removal of apoptotic bodies. A subset of the 29 renal biopsies used to measure ecDNA labeled (*n* = 6, the biopsies with sample remaining) with an anti-human DNase I antibody demonstrated patients with MPO-ANCA GN patients had less DNase I expression within the tubulointerstitium and glomeruli when compared with those from control patients with MCD (*n* = 6) (Figure 1C). Semiquantification of both ecDNA and DNase I deposition in the kidney demonstrated a significant difference in both ecDNA and DNase I (Figure 1, B–E).

### Enhanced deposition of ecDNA and reduced expression of DNase I in the kidneys of mice with anti-MPO GN are attenuated with exogenous DNase I administration.

**Figure 2:**
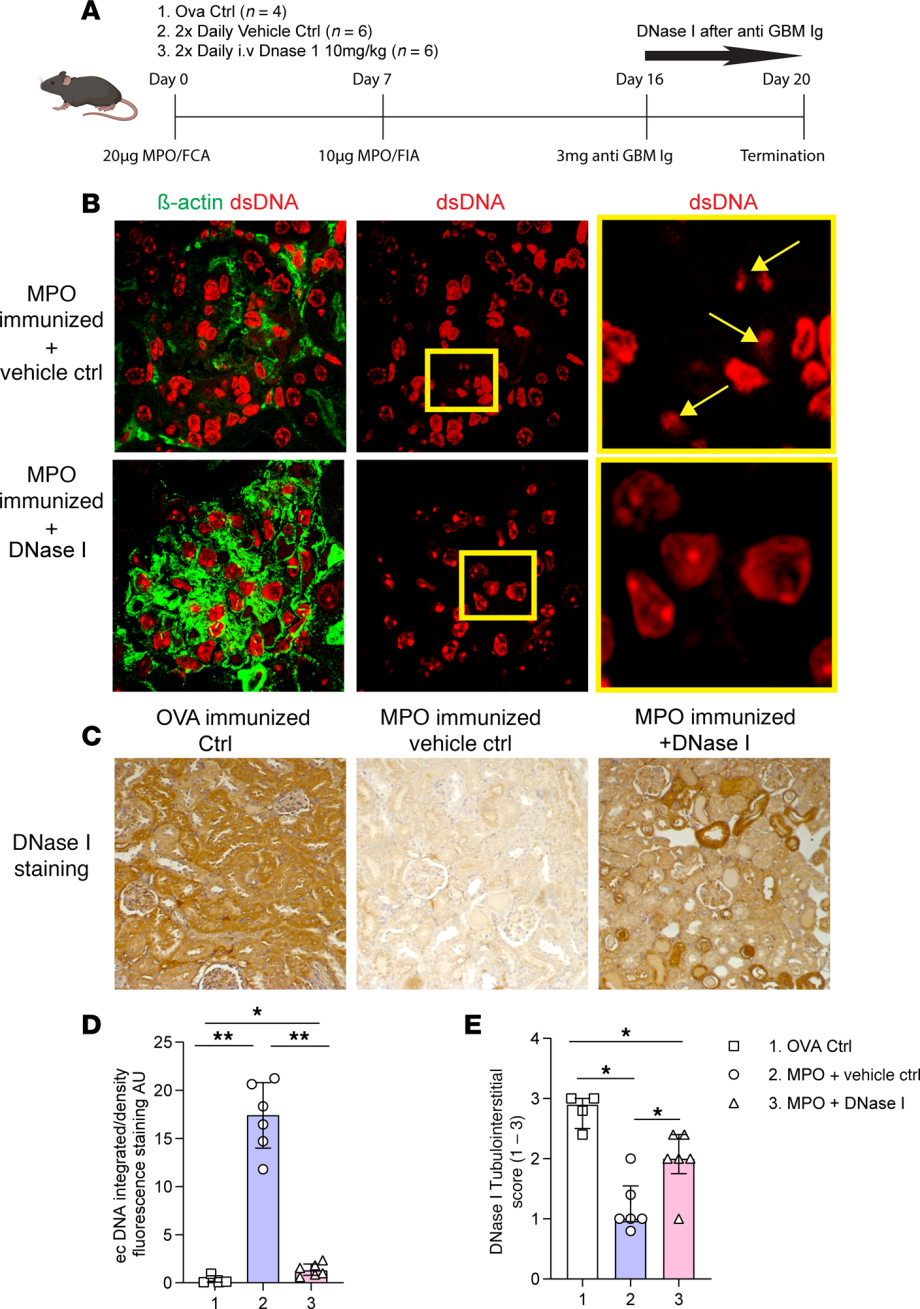
Mice with autoimmune anti-MPO GN have significantly enhanced renal deposition of ecDNA and diminution of DNase I, which is restored by iv rhDNase I. (**A**) Experimental timeline outlining the induction of anti-MPO GN via immunization and subnephritogenic dose of anti-GBM Ig with OVA (*n* = 4) control (irrelevant antigen), MPO-immunized and vehicle-treated control (*n* = 6) and MPO immunized, treated with DNase I (*n* = 6). (**B**) MPO-immunized vehicle-treated mice or MPO-immunized mice that were later treated with ivDNase I. Sections were stained for ecDNA (red) and counterstained with β-actin (green). (**C**) Representative images of DNase I immunohistochemistry across experimental groups. (**D**) Semiquantitation of ecDNA in kidney sections made from OVA (1, squares), MPO-immunized vehicle-treated (2, circles), and MPO-immunized ivDNase I–treated groups (3, triangles) was performed using ImageJ (NIH), and the amount of ecDNA is expressed as arbitrary units per glomerular cross section (AU/gcs). (**E**) Histological scoring of the tubulointerstitium of mouse kidneys for DNase scored relative to the intensity of staining observed (with 1 being equivalent to MPO vehicle Ctrl treated, 2 being equivalent to image depicting MPO-immunized+DNase I, and 3 reflected in the image of the OVA Ctrl mice). *P < 0.05, **P < 0.01. Data are median (IQR) from 6 mice in each group analyzed by Kruskal-Wallis, for 3 or more groups. Original magnification, 400×. IQR, interquartile range; MPO, myeloperoxidase; GN, glomerulonephritis; Ctrl, control; DNase I, deoxyribonuclease I; ecDNA, extracellular deoxyribonucleic acid; dsDNA, double-stranded deoxyribonucleic acid; OVA, ovalbumin.

Using an established murine model of anti-MPO GN (38), we tested the hypothesis that disease is associated with increased levels of ecDNA deposition in murine kidneys. Mice were immunized with MPO, and GN was induced with anti–glomerular basement membrane globulin (anti-GBM Ig) (Figure 2A). Glomerular injury in this model of active anti-MPO autoimmunity is mediated by cellular effectors, and OVA-immunized mice injected with anti-GBM globulin serve as negative controls. Thus, this model shows the same pattern of injury with respect to ecDNA and decreased DNase I expression as observed in the human disease (in previous Figure 1). To test whether the reductions in endogenous DNase I levels could be offset using exogenous DNase I to alleviate clinical endpoints of disease, anti-MPO GN mice were given twice daily injections of human DNase I for 4 days. This treatment substantially attenuated glomerular ecDNA deposition (Figure 2, B and C) and increased renal expression of DNase I when compared with control animals (Figure 2, B–E). Naive C57BL/6J mice demonstrated minimal ecDNA deposition in glomeruli in comparison (Supplemental Figure 1A). As expected, endogenous DNase I levels were unaffected in OVA-immunized control mice (Figure 2C).

### Exogenous DNase I treatment diminishes glomerular injury and glomerular NET formation in MPO-ANCA GN.

**Figure 3:**
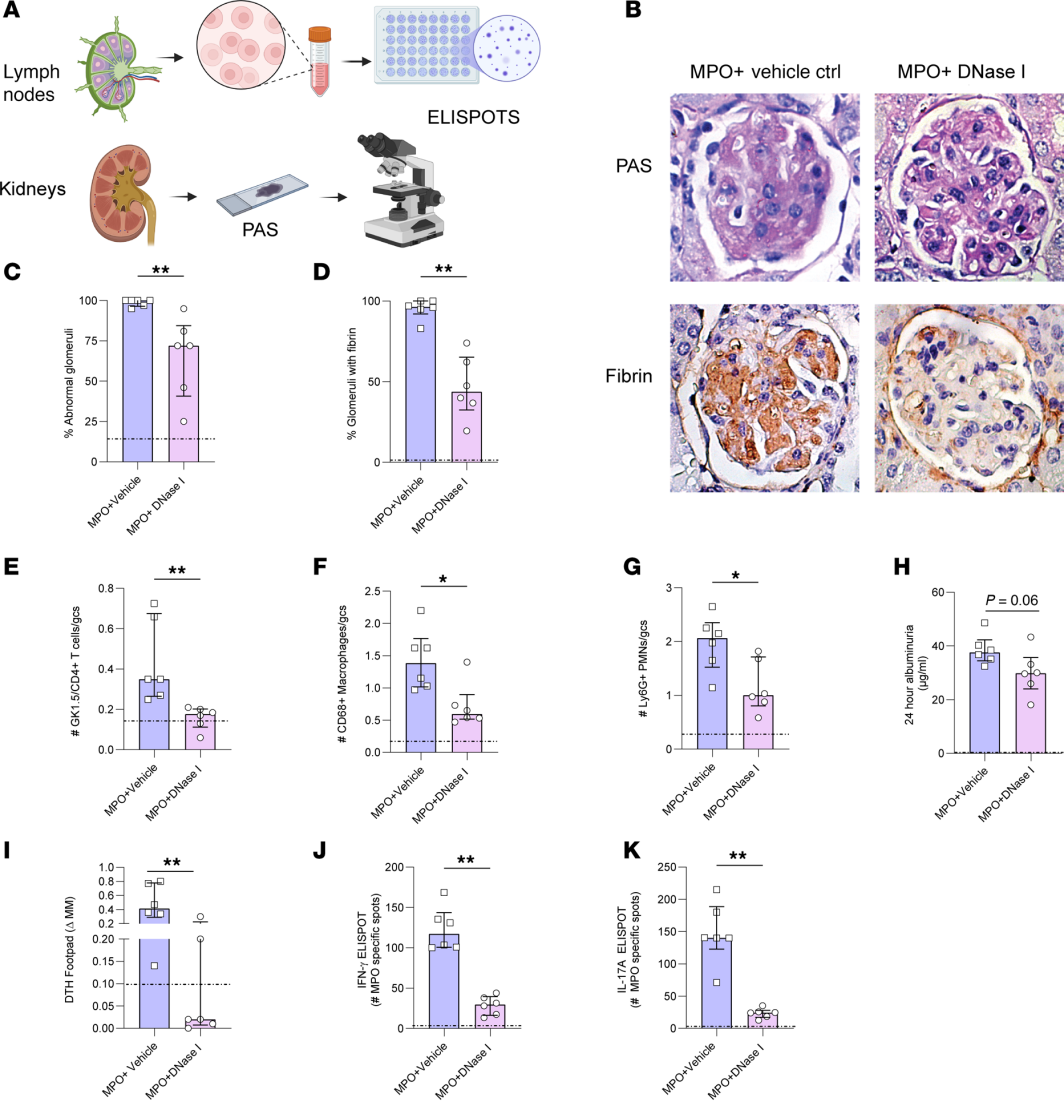
rhDNase I treatment reduces histological glomerular injury, leukocyte recruitment, and functional injury in experimental MPO-ANCA GN. (**A**) Experimental endpoints. Animals were assessed for the following parameters: murine kidney sections stained with (**B**) periodic acid–Schiff and fibrin staining of mice treated with vehicle control versus DNase I; (**C**) percentage of abnormal glomeruli after the assessment of (**D**) percentage of glomeruli containing fibrin. (**E**) Immunoperoxidase staining for glomerular infiltrating leukocytes, Gk1.5^+^CD4^+^ T cells, (**F**) CD68^+^ macrophages, and (**G**) GR1^+^ neutrophils. (**H**) Twenty-four-hour albuminuria. Animals were also assessed for (**I**) dermal delayed type hypersensitivity (DTH) response after intradermal MPO injection and (**J**) frequency of MPO-specific IFN-γ– and (**K**) frequency of MPO-specific IL-17A–producing cells in lymph nodes draining sites of MPO immunization. *P < 0.05, **P < 0.01. Data are median (IQR) from 6 mice in each group analyzed by a Mann-Whitney *U* test. Dotted line represents the OVA-immunized reference control. Original magnification, 600×. MPO, myeloperoxidase; DNase I, deoxyribonuclease I; Ctrl, control; IQR, interquartile range.

Having demonstrated that DNase I cleared ecDNA and maintained homeostasis of endogenous renal DNase I, further endpoints were assessed to determine the effect of enzyme administration on histological and functional injury and renal inflammation (Figure 3A, experimental endpoints). DNase I–treated mice exhibited less glomerular injury as seen by lower proportions of abnormal glomeruli (Figure 3, B and C), with less incidence of glomerular crescents, segmental necrosis, glomerular expansion, and cell infiltration (Supplemental Figure 2, A–D); less fibrin deposition (Figure 3, B and D); and fewer glomerular leukocytes when compared with saline control mice that developed anti-MPO GN (Figure 3, F–H). The mean albuminuria over 24 hours was numerically lower in DNase I–treated mice, but this did not reach statistical significance (*P* = 0.06, Figure 3H). DNase I was administered to mice 16 days after establishment of autoimmunity, and GN was initiated with anti-GBM Ig. Despite only 4 days of treatment, DNase I–treated mice had a significant reduction in dermal delayed type hypersensitivity (DTH), 24 hours after injecting the footpad with MPO, compared with the saline-treated control group (Figure 3I). A significant decrease in MPO-specific IFN-γ– and IL-17A–producing cells from lymph node–draining sites of MPO immunization was observed in the DNase I–treated group (Figure 3, J and K). As expected OVA-immunized control mice did not develop anti-MPO autoimmunity as seen by the absence of GN and abnormal glomeruli and reduced leukocyte accumulation in the glomeruli (Figure 3, A–K).

**Figure 4:**
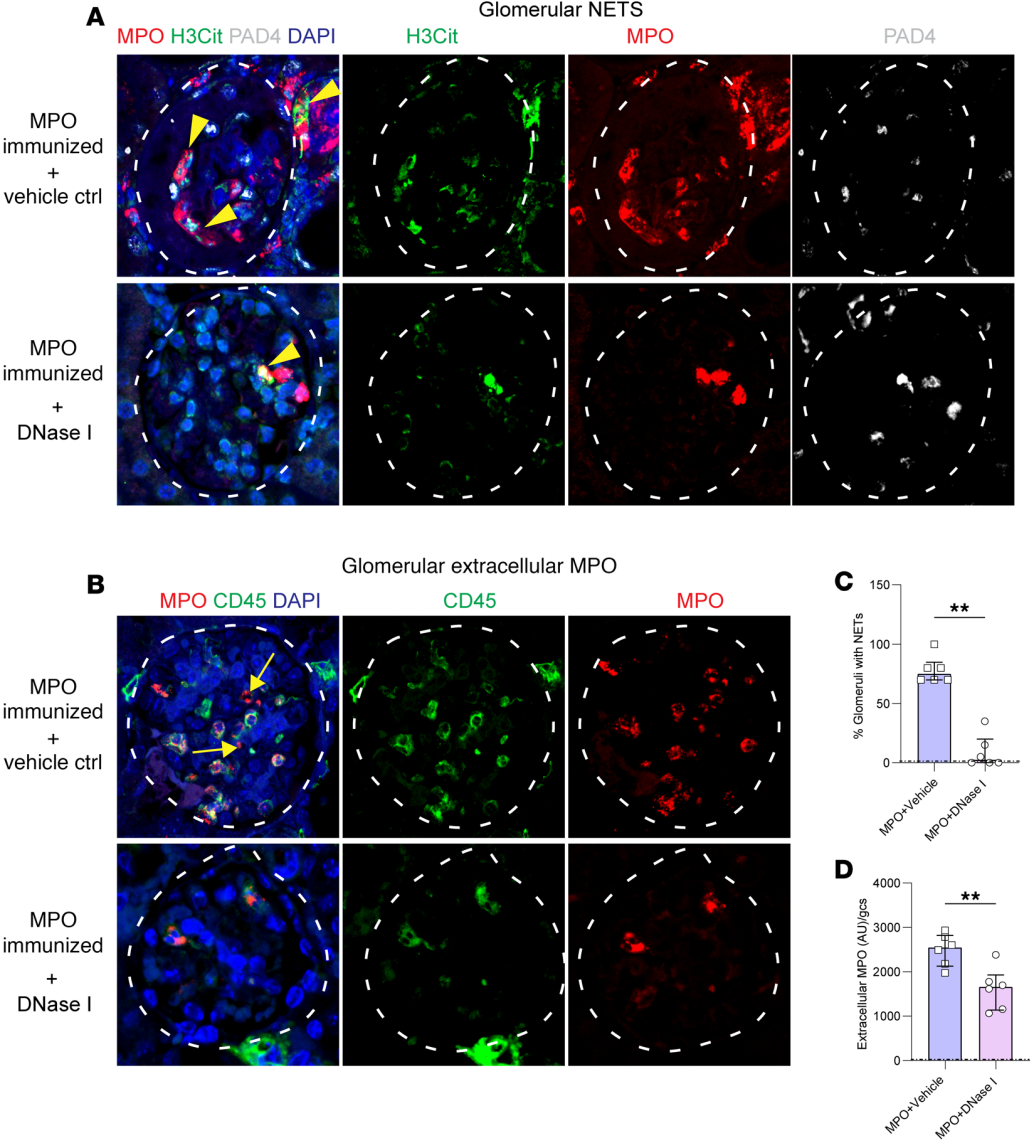
DNase I treatment reduces NET formation and deposition of extracellular MPO in established anti-MPO-ANCA GN. (**A**) Kidney sections from MPO-ANCA GN mice receiving either vehicle control or ivDNase I treatment stained for NETs by colocalization of MPO (red), H3Cit (green), PAD4 (white), and DAPI (blue), indicated by yellow arrowheads appearing as magenta/yellow-colored areas of staining. (**B**) Kidney sections labeled for MPO (red), CD45 (green), and DAPI (blue) were assessed for intracellular MPO (colocalization of CD45 and MPO appears yellow, whereas extracellular MPO [noncolocalized CD45 and MPO] appears as red only, indicated by arrows). (**C**) NET quantitation in glomeruli of ivDNase I–treated and control MPO-ANCA GN mice. (**D**) Extracellular MPO quantitation in glomeruli of ivDNase I–treated and control MPO-ANCA GN animals. **P < 0.01. Data are median (IQR) from 6 mice in each group analyzed by Mann-Whitney *U*. Dotted line represents the OVA-immunized reference control. Original magnification, 600×. MPO, myeloperoxidase; DNase I, deoxyribonuclease I; Ctrl, control; PAD4, peptidylarginine deiminase 4; H3Cit, citrullinated histone 3; IQR, interquartile range.

We then sought evidence of the therapeutic efficacy of recombinant human (rh) DNase I to reduce the number of NETs depositing MPO in the glomeruli of kidneys of mice with anti MPO-GN. In the control saline-treated mice NETs were found in 80% of glomeruli, while DNase I treatment markedly reduced the proportion of glomeruli with NETs to less than 10% (Figure 4A). This reduction in NETs was associated with less extracellular MPO deposition (Figure 4B). Semiquantification demonstrated that rhDNase I treatment significantly reduced the number of glomeruli with NETs and extracellular MPO (Figure 4, C and D).

### Exogenous DNase I attenuates the development of anti-MPO autoimmunity.

**Figure 5:**
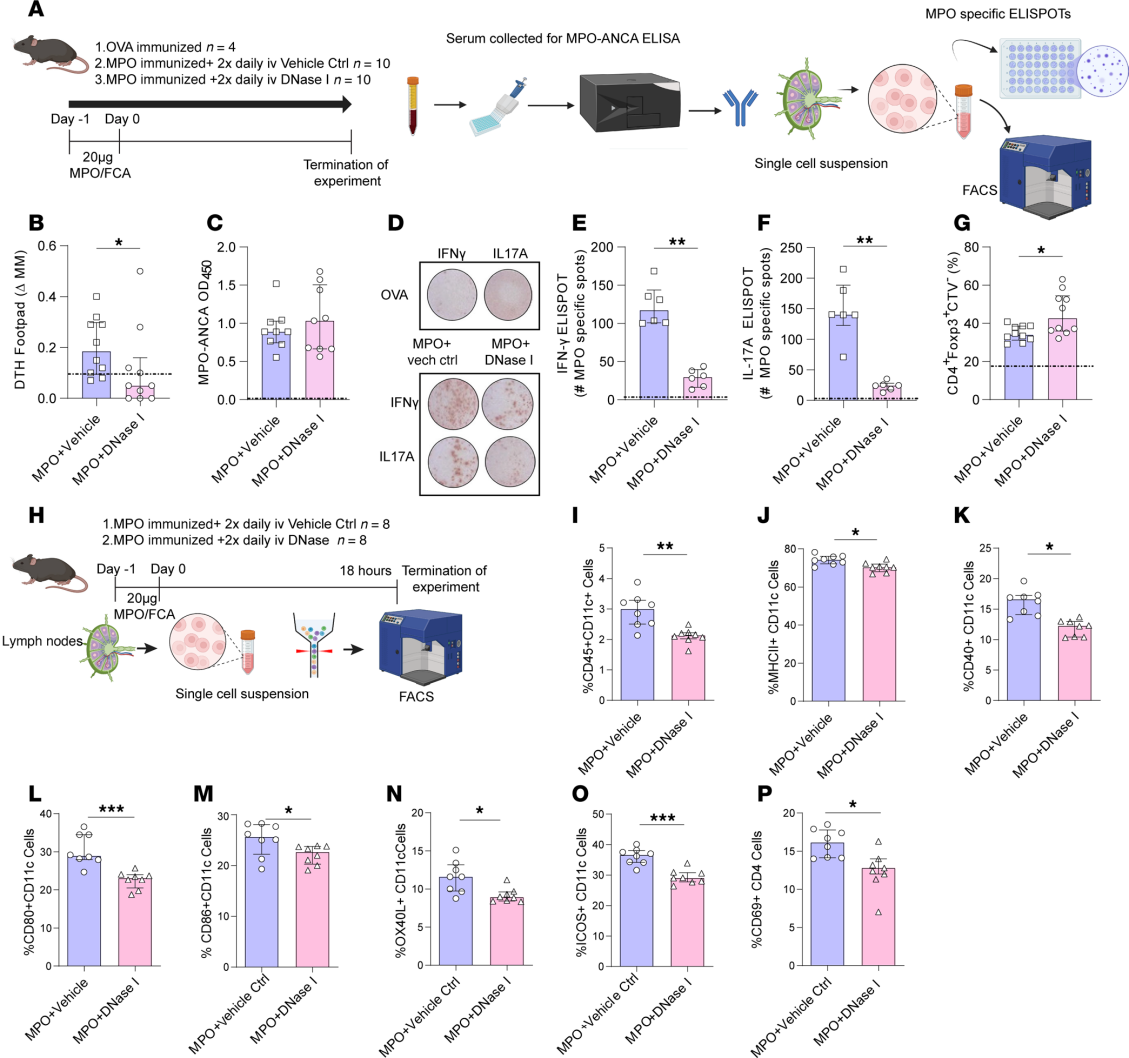
DNase I treatment reduces autoimmunity to MPO and reduces DC and T cell activation from the draining lymph nodes. (**A**) Experimental timeline for a 10-day MPO immunization model where mice receive vehicle ctrl or ivDNase I 1 day prior to immunization with MPO. Mice subsequently receive vehicle ctrl or ivDNase I daily until the end of the experiment at 10 days postimmunization. Animals were assessed for (**B**) delayed type hypersensitivity response after intradermal MPO injection, (**C**) serum MPO-ANCA and frequency of (**D**) MPO-specific IFN-γ and MPO-specific IL-17A via ELISPOTs quantitated in **E** and **F**, and number of ex vivo CD4^+^FoxP3^+^CTV^–^ lymphocytes that proliferate in response to MPO, from lymph nodes draining sites of MPO immunization (**G**). (**H**) Experimental timeline for an 18-hour MPO immunization model where mice receive vehicle ctrl or ivDNase I 1 day prior to immunization with MPO, with experiment terminated 18 hours postimmunization. Lymph nodes draining the site of MPO immunization were assessed for the frequency of (**I**) CD11c DCs that also expressed (**J**) MHCII, (**K**) CD40, (**L**) CD80, (**M**) CD86, (**N**) OX40L, or (**O**) ICOS. (**P**) Frequency of activated CD4^+^ T cells in lymph nodes draining the site of MPO immunization. *P < 0.05, **P < 0.01, ***P < 0.001, analyzed by Mann-Whitney *U* test. Dotted line represents the OVA-immunized reference control. Data are median (IQR) from *n* = 10 mice in the 10-day model and *n* = 8 mice in the 18-hour model. CTV, cell tracker violet; D-1, day minus 1; FCA, Freund’s complete adjuvant; IV, intravenous tail vein injection; DTH, delayed type hypersensitivity; MPO, myeloperoxidase; IFN-γ, interferon-gamma; IL-17A, interleukin-17A; ELISPOT, enzyme-linked immunoSpot; ICOS, inducible T cell co-stimulator; MHCII, major histocompatibility complex class II; DNase I, deoxyribonuclease I; Ctrl, control; IQR, interquartile range.

To assess the effect of exogenous rhDNase I on the development of anti-MPO autoimmunity, DNase I or vehicle control was administered to C57BL/6J WT mice (*n* = 10 each group) 1 day prior to the groups receiving subcutaneous immunization with rmMPO in FCA, followed by daily injections of saline or rhDNase I for a further 10 days (Figure 5A). When compared with saline control mice, rhDNase I–treated mice had a significant reduction in dermal DTH to MPO (Figure 5B), but serum MPO-ANCA titers were unchanged between the vehicle-treated and rhDNase I–treated groups (Figure 5C). Ex vivo MPO stimulation of cells from the lymph nodes that drain immunization sites demonstrated that rhDNase I reduced the numbers of IFN-γ–secreting cells detected via ELISPOT (Figure 5D) whereas IL-17A production was unaltered (Figure 5, E and F). Moreover, draining lymph node cells restimulated ex vivo with MPO for 72 hours proliferated in response to exposure to their cognate antigen as indicated by a significant increase in CD4^+^FoxP3^+^CTV^–^ via FACS (Figure 5G). Collectively, these data strongly indicate that rhDNase I inhibits the development of anti-MPO autoimmunity by reducing the frequency of Th1 MPO-specific effector cells while increasing the frequency of MPO-specific Tregs with increased capacity to respond to MPO.

### Exogenous DNase I inhibits DC migration and activation in lymph nodes draining MPO immunization sites.

To test the hypothesis that rhDNase I modulates DC activation after MPO immunization, we examined the effects of DNase I at the time of antigen presentation. C57BL/6J mice received ivDNase I 24 hours before immunization with rmMPO in FCA, and experiments ended 18 hours later (Figure 5H). Draining lymph nodes were removed and CD11c^+^ cells examined by flow cytometric analysis for coexpression of activation markers. Eighteen hours after immunization with MPO, the mice treated with ivDNase I had a significant reduction in the proportion of CD11c^+^ cells (Figure 5I). Also, DCs migrating to the draining lymph nodes showed a reduction in the expression of activation and costimulation markers MHC class II, CD40, CD80, CD86, OX40L, and ICOS (Figure 5, J–O). Mice treated with ivDNase I also had a significant reduction in the proportion of activated T cells at this early time point (CD4^+^CD69^+^, Figure 5P). These findings are consistent with DNase I inhibition of DC activation and migration to draining lymph nodes.

### DNase I attenuates disease in experimental GN induced by transfer of anti-MPO antibodies.

**Figure 6:**
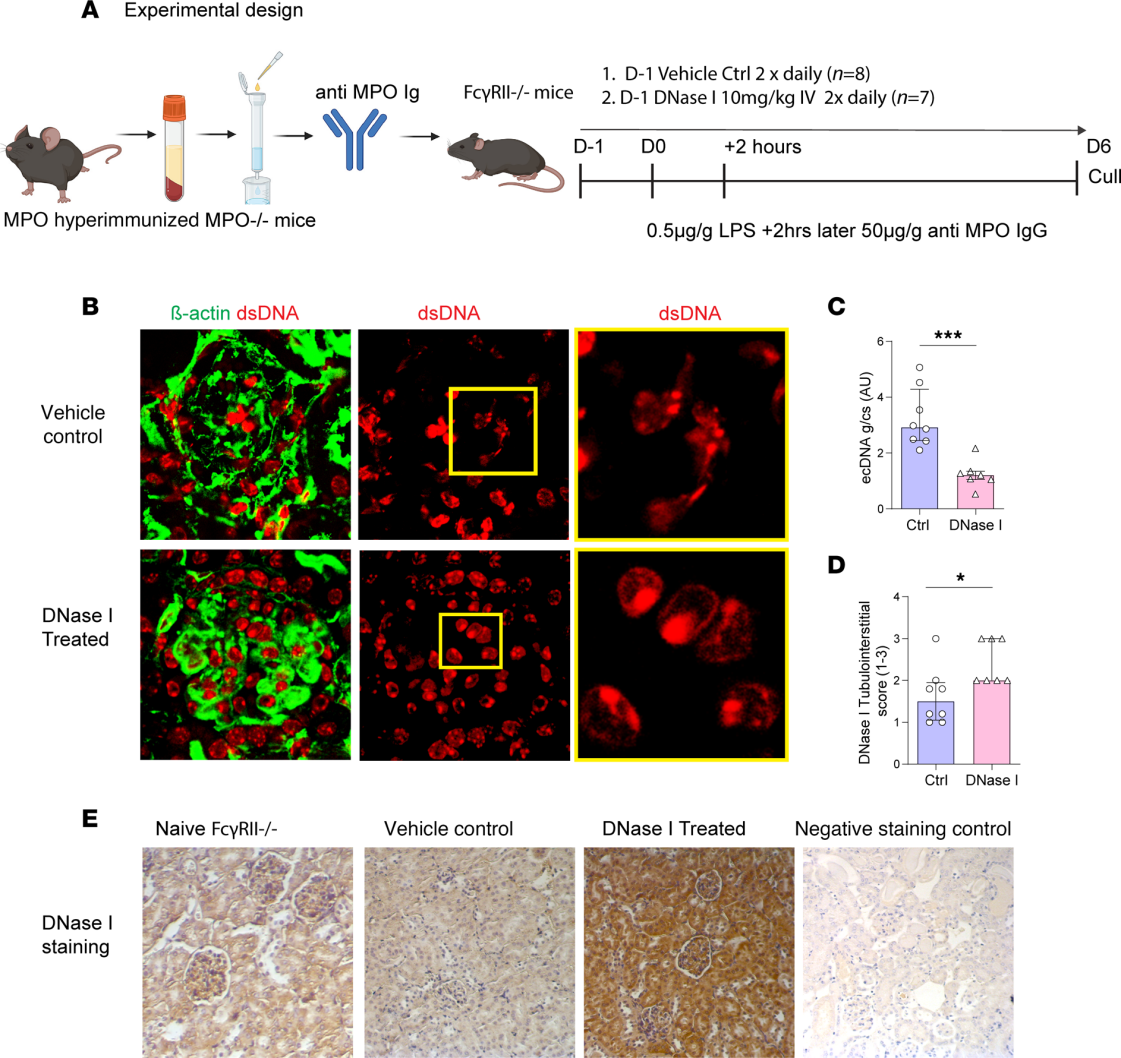
EcDNA is reduced in DNase I–treated mice with GN induced by passive transfer of anti-MPO antibodies. (**A**) Experimental timeline, where mice receive vehicle control or ivDNase I 1 day prior to receiving LPS and anti-MPO IgG. (**B**) Kidney sections from mice receiving either vehicle control or ivDNase I treatment stained for DNA (red) and β-actin (green) were quantitatively assessed using ImageJ (NIH) for (**C**) ecDNA (expressed in AU) per glomerular cross section. (**D**) Histological scoring of the tubulointerstitium of mouse kidneys for DNase I was performed on (**E**) kidney sections immunohistochemically labeled for DNase I. *P < 0.05, ***P < 0.001. Data are median (IQR) from 8 mice in the control group and 7 mice in the treatment group analyzed by Mann-Whitney *U*. Original magnification, 400×. Ctrl, control; DNase I, deoxyribonuclease I; ecDNA, extracellular deoxyribonucleic acid; IQR, interquartile range.

**Figure 7:**
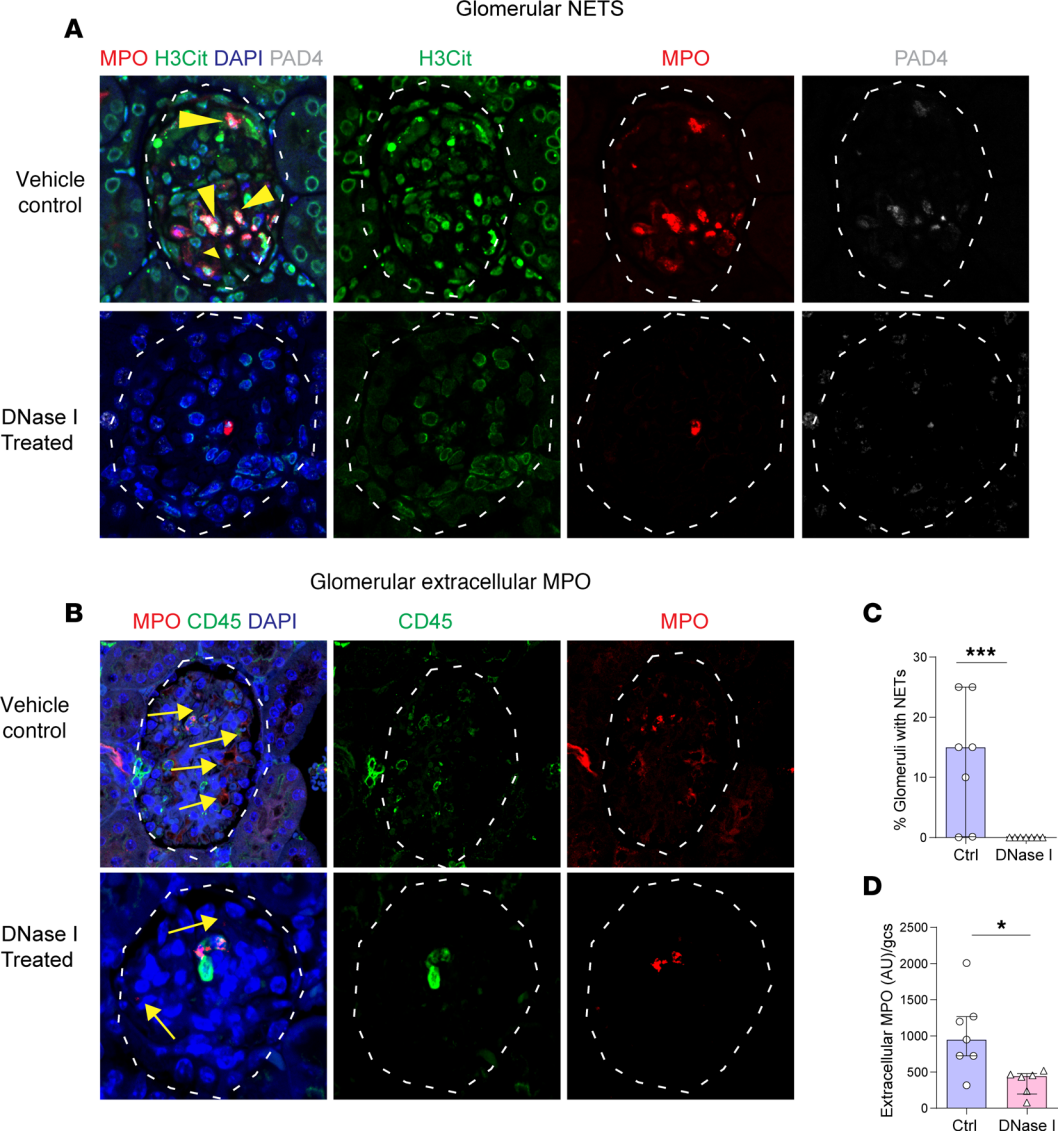
NET formation is abolished by DNase I treatment in a passive ANCA-dependent model of anti-MPO GN. (**A**) Kidney sections from passive ANCA-MPO GN mice receiving either vehicle control or ivDNase I treatment stained for NETs as seen by colocalization of MPO (red), H3Cit (green), PAD4 (white), and DAPI (blue) indicated by arrows as bright magenta/white colocalization. (**B**) Kidney sections labeled for MPO (red), CD45 (green), and DAPI (blue) were assessed for intracellular MPO (colocalizations of CD45 and MPO) and extracellular MPO (noncolocalized CD45 and MPO, indicated as red staining alone, as indicated by arrows). (**C**) NET quantitation in glomeruli of ivDNase I–treated and control passive ANCA-MPO GN animals. (**D**) Extracellular MPO quantitation in glomeruli of ivDNase I–treated and control passive ANCA-MPO GN animals. *P < 0.05, ***P < 0.001. Data are median (IQR) from 8 mice in the control group and 7 in the treatment group analyzed by Mann-Whitney *U*. Original magnification, 600×. MPO, myeloperoxidase; PAD4, peptidyl arginine deiminase 4; H3Cit, citrullinated histone 3; Ctrl, control; DNase I, deoxyribonuclease I; IQR, interquartile range.

**Figure 8:**
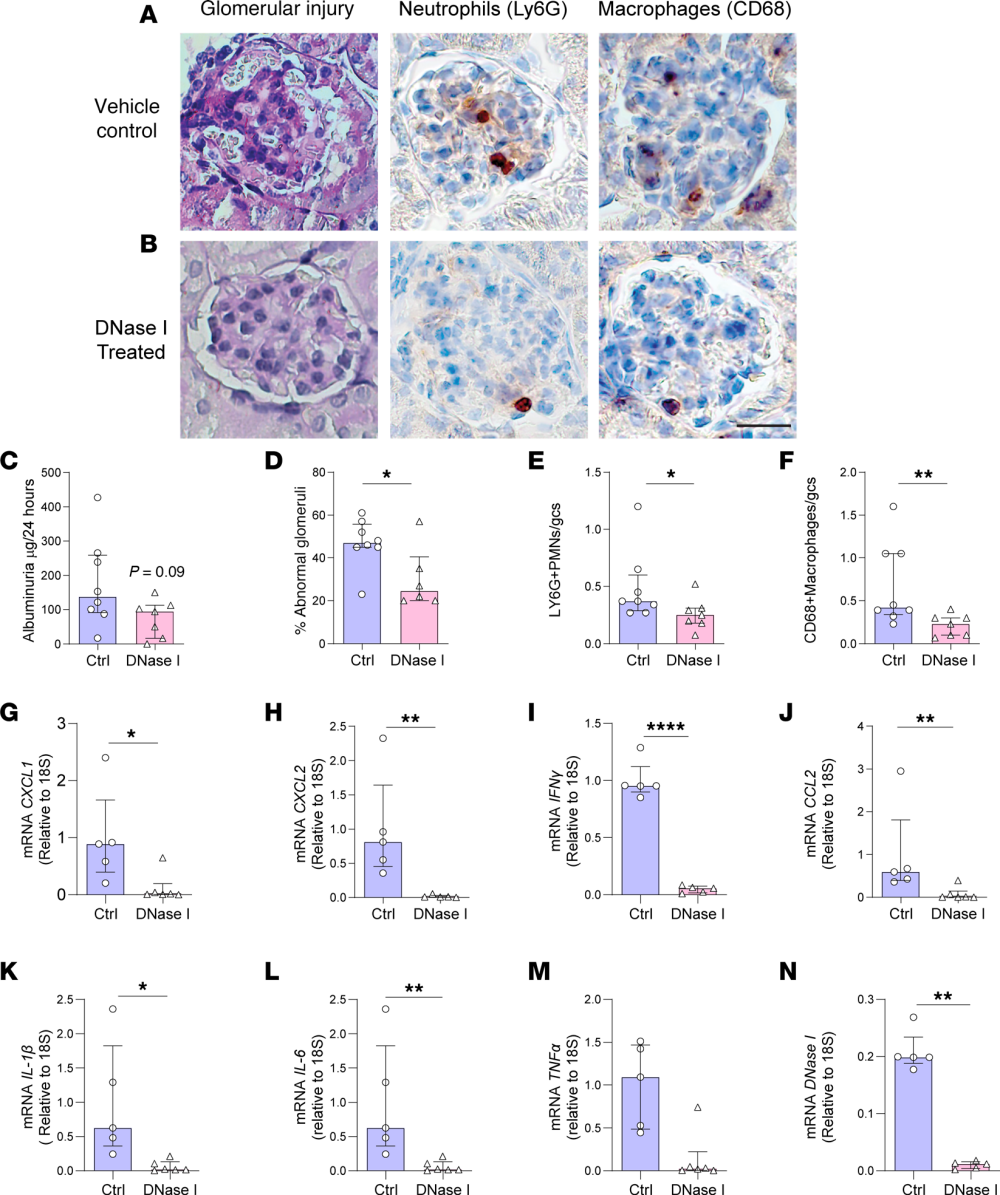
DNase I treatment reduces glomerular injury and inflammatory gene expression in the kidney of mice in an ANCA-mediated model of anti-MPO GN. Kidney sections from animals receiving saline control or DNase I treatment after passive ANCA-induced anti-MPO GN were enumerated for evidence of functional glomerular injury as seen by (**A** and **B**) abnormal glomeruli, polymorphonucleocyte infiltration and macrophage infiltration, and (**C**) 24-hour albuminuria levels. (**D**–**F**) Semiquantification of histological damage and leukocyte infiltration. RNA was extracted from the kidneys of animals receiving saline control or DNase I treatment after passive ANCA-induced anti-MPO GN and assessed by qRT-PCR for (**G**) *CXCL1*, (**H**) *CXCL2*, (**I**) *IFN-γ*, (**J**) *CCL2*, (**K**) *IL-1β*, (**L**) *IL-6*, (**M**) *TNFα*, (**N**) *DNase I*. Values are normalized to 18S ribosomal RNA. *P < 0.05, **P < 0.01, ****P < 0.0001. Data are median (IQR) from 8 mice in the control group and 7 mice in the treatment group analyzed by Mann-Whitney *U*. Original magnification, 400×. qRT, quantitative real time; Ctrl, control; DNase I, deoxyribonuclease I; IQR, interquartile range.

As the International Society of Nephrology recommends therapeutic drug testing be undertaken in multiple preclinical animal models (39), we utilized a second animal model of anti-MPO GN. As anti-MPO antibodies are important in activating neutrophils in MPO-ANCA GN, the therapeutic efficacy of DNase I shown in the 20-day T cell–mediated model of anti-MPO GN was also tested in a model of anti-MPO GN induced by transfer of anti-MPO antibodies into mice primed with LPS (40). DNase I (or vehicle) was given 24 hours prior to disease induction and every 12 hours until the end of the experiment (Figure 6A). Renal ecDNA deposition was significantly less (Figure 6, B and C, *P* < 0.05) and DNase I expression was significantly greater in ivDNase I–treated mice than in vehicle-treated mice with GN (Figure 6, D and E, *P* < 0.05). Glomerular NET formation (Figure 7A) and deposition of extracellular MPO (Figure 7B) were both significantly greater (*P* < 0.05) in control vehicle-treated mice than in animals that received ivDNase I (Figure 7, C and D). Histological assessment of glomerular injury demonstrated ivDNase I treatment prevented the development of abnormal glomeruli and infiltrating immune cells (Figure 8, A and B). As in the 20-day active model, there were a reduction in albuminuria that did not reach a level of significance (Figure 8C), a reduced number of abnormal glomeruli defined as a reduction in segmental necrosis and infiltration of cells into Bowman’s space as described previously (Figure 8D), and reduced numbers of glomerular infiltrating Ly6G^+^ neutrophils (Figure 8E) and CD68^+^ macrophages (Figure 8F). Analysis of kidney mRNA for common inflammatory markers demonstrated significant diminution of chemokines that control neutrophil recruitment (*CXCL1* and *CXCL2*) (Figure 8, G and H) and macrophage recruitment (*IFNγ* and *CCL2*) and markers of inflammation, including *IL-1β*, *IL-6*, and *TNFα* (Figure 8, I–M). Analysis of *DNase I* mRNA showed that nontreated diseased mice had significantly more intrarenal *DNase I* gene expression than the ivDNase I–treated group, suggesting increased *DNase I* gene transcription in response to inflammation, whereas the DNase I group, which had fewer markers of inflammation, also had reduced levels of *DNase I* expression (Figure 8N). Collectively, the data from 2 different and complementary models of anti-MPO GN indicate that ecDNA deposition can be cleared by exogenous DNase I, resulting in significantly attenuated glomerulonephritis.

### AAV delivery of DNase I has enhanced therapeutic efficacy over ivDNase I to reduce inflammation in MPO-ANCA GN.

**Figure 9:**
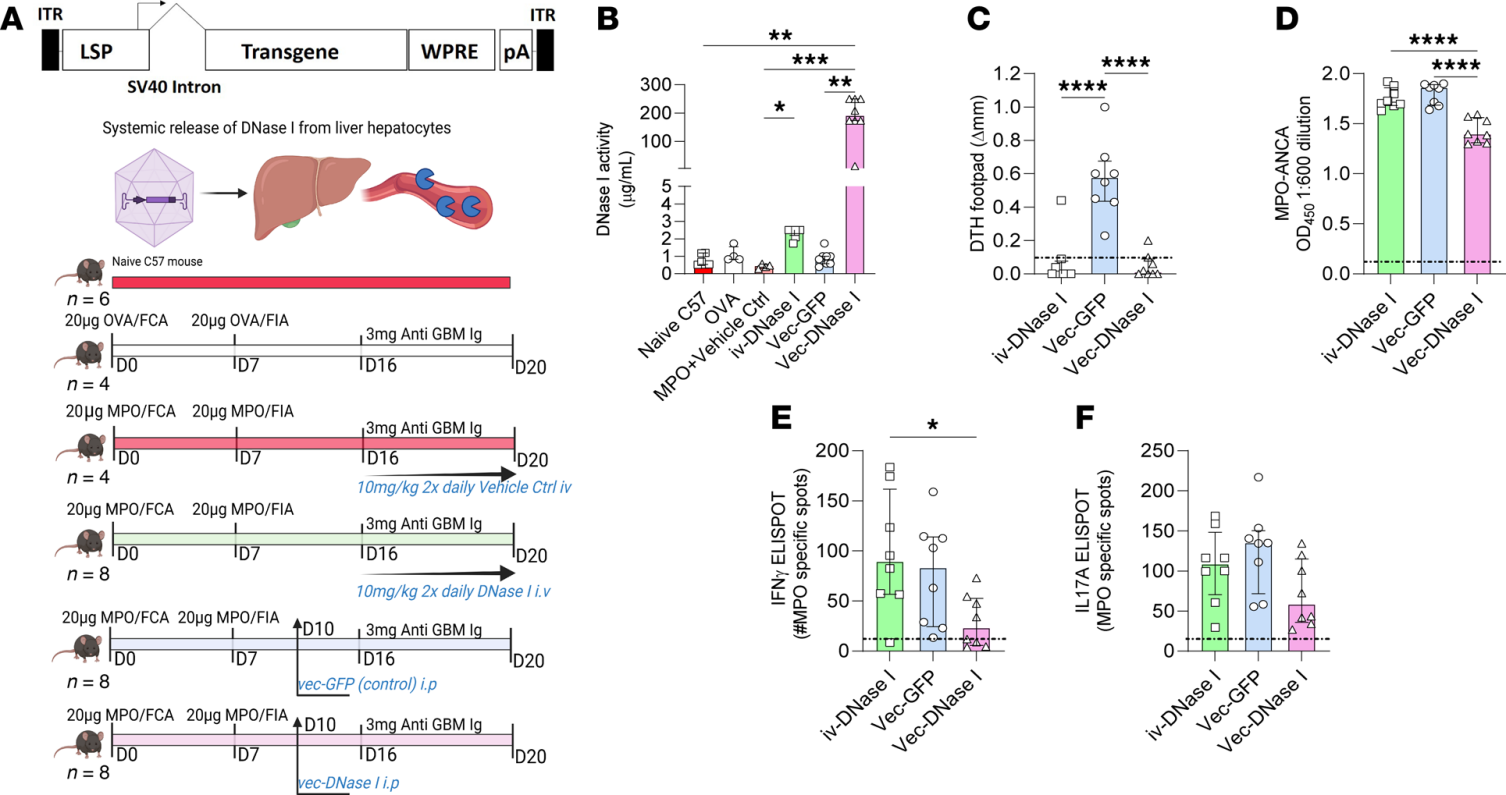
vec-DNase I treatment is superior to exogenously administered rhDNase I in reducing autoimmunity to MPO in the 20-day ANCA anti-MPO GN model. (**A**) Vector constructs used in the 20-day ANCA model and treatment strategy. (**B**) Serum DNase I activity levels, including naive and OVA-immunized mice as a comparator. (**C**) After completion of the experiment, mice were assessed for footpad delayed type hypersensitivity response in response to MPO, (**D**) MPO-ANCA levels, and the frequency of anti-MPO–reactive cells in the lymph nodes that drained the site of MPO immunization that secreted (**E**) IFN-γ or (**F**) IL-17A. *P < 0.05, **P < 0.01, ***P < 0.001, ****P < 0.0001. Data are median (IQR) from *n* = 8 mice per group analyzed by Kruskal-Wallis test with Dunn’s post hoc test for multiple comparisons. Dotted line represents OVA-immunized mice as a reference control. vec, adeno-associated viral vector; Ctrl, control; DNase I, deoxyribonuclease I; FCA, Freund’s complete adjuvant, FIA, Freund’s incomplete adjuvant; GFP, green fluorescent protein; H3Cit, citrullinated histone 3; ITR, intron; IQR, interquartile range; LSP, liver-specific promoter; MPO, myeloperoxidase; pA, polyadenylation sequence; WPRE, woodchuck hepatitis virus posttranscriptional regulatory element.

**Figure 10:**
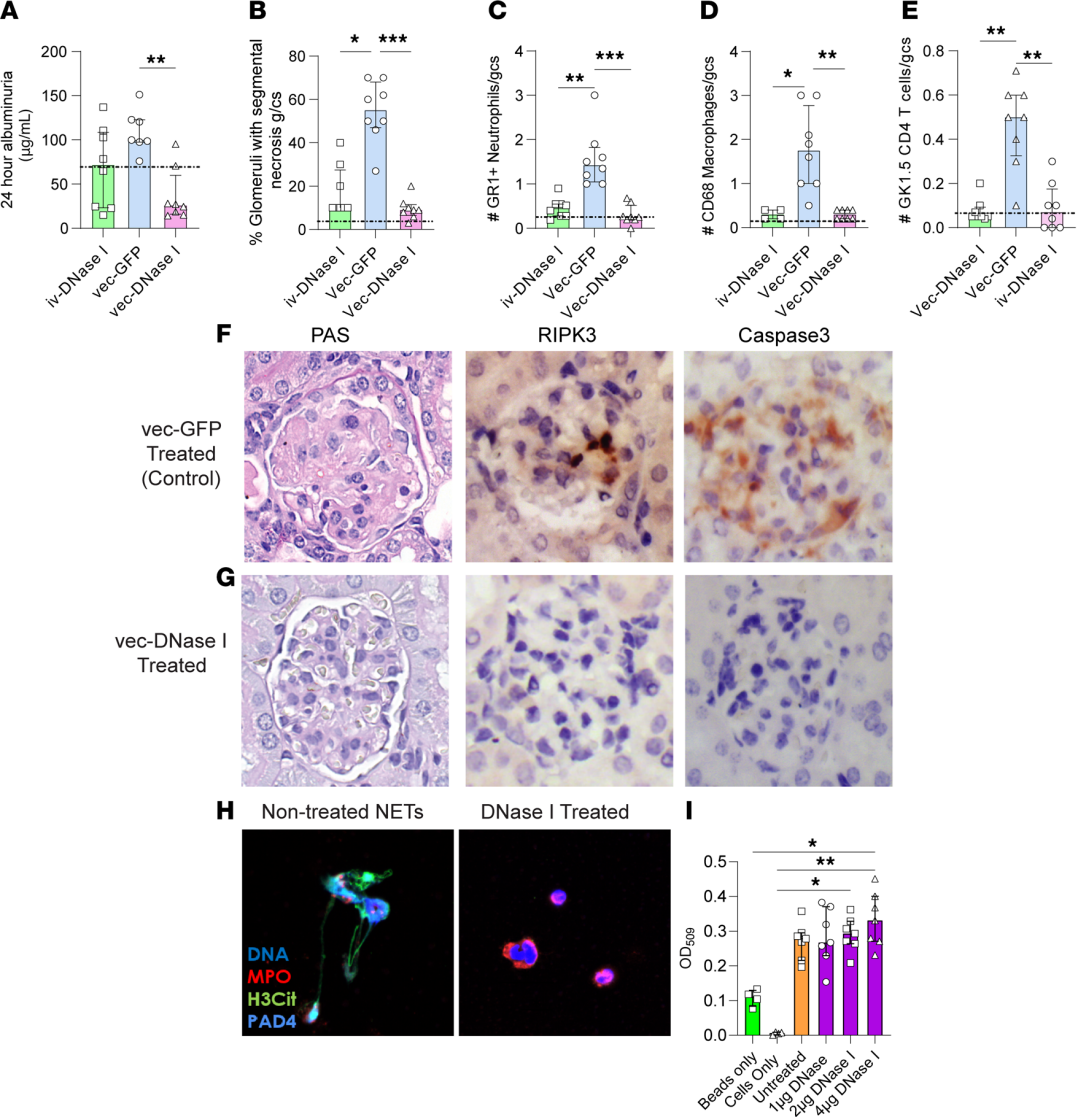
vec-DNase I treatment reduces kidney injury, glomerular leukocyte infiltration, cell death, and NETosis. Treatment with vec-DNase I significantly reduces 24-hour albuminuria (**A**) and histological glomerular injury compared with the control GFP vector (**B**). Glomerular leukocyte recruitment is reduced in mice treated with both the exogenous DNase I and vec-DNase I when compared with the control AAV-GFP vector (**C**–**E**). Both segmental necrosis and markers of cell death RIPK3 and caspase-3 are reduced in mice treated with the vec-DNase I compared with the control vec-GFP (**F** and **G**). NET assays stimulated with PMA and treated with DNase I at 1 μg/mL demonstrate attenuation of NETs, with DNA in blue, MPO in red, H3Cit in green, and PAD4 in gray/blue (**H**). pHrodo Green *S*. *aureus* bioparticles were used for phagocytosis assays with neutrophils incubated with DNase I at increased concentrations 0–4 μg/mL (purple bars) and compared with untreated neutrophils (orange bar) to show that DNase I does not inhibit neutrophil phagocytosis (**I**). *P < 0.05, **P < 0.01, ***P < 0.001. In vivo animal data are median (IQR) from 8 mice in each group analyzed by Kruskal-Wallis test with Dunn’s post hoc test for multiple comparisons. Dotted line represents OVA-immunized mice as a reference control. In vitro phagocytosis data expressed as the average of duplicate wells with data expressed as the median (IQR). Dotted line represents bead-only reference control. RIPK3, receptor-interacting serine/threonine-protein kinase 3; vec, adeno-associated viral vector; Ctrl, control; DNase I, deoxyribonuclease I; GFP, green fluorescent protein; H3Cit, citrullinated histone 3; IQR, interquartile range; MPO, myeloperoxidase; PAD4, peptidyl arginine deiminase 4; PMA, phorbal myristate acetate.

A limitation of the therapeutic potential of DNase I is that it has a half-life of only less than 5 hours. The iv-rhDNase I treatment of the MPO-ANCA required twice daily doses to compensate for short serum half-life of the enzyme. We investigated whether delivery of murine DNase I could be facilitated using an AAV to systemically deliver recombinant murine (rm) DNase I (vec-DNase I, Figure 9A, experimental plan). In our previous study, we have shown that the liver provides a continuous superphysiological supply of DNase I in the circulatory system within 3 days of vector injection that stably persists for at least 6 months (41). Using our anti-MPO GN model where disease is mediated by active autoimmunity to MPO, we gave a single dose of vec-DNase I or vec-GFP 10 days after the establishment of anti-MPO autoimmunity (Figure 9A). This resulted in an approximately 200-fold increase in DNase I activity in serum compared with either ivDNase I delivered twice daily or vec-GFP control-treated animals, whereas naive C57BL/6J mice, OVA control, and MPO-immunized mice had minimal amounts of DNase I activity (Figure 9B). Western blot analysis of DNase I levels in the serum showed significant levels of rhDNase I in the iv-treated groups and significant rmDNase I in mice treated with the vec-DNase I (Supplemental Figure 3, A–C). Western blot detection of DNase I in kidney lysate failed to detect any substantial level of DNase I (Supplemental Figure 3D). vec-DNase I significantly reduced DTH similarly to ivDNase I (Figure 9C). Interestingly, vec-DNase I reduced the generation of MPO-ANCA whereas ivDNase I had no effect on MPO-ANCA levels (Figure 9D). vec-DNase I significantly reduced the number of both IFN-γ and IL-17A MPO-specific effector cells in the draining lymph nodes (Figure 9, E and F, similar to effects seen with ivDNase I administration in the previous sections). Importantly, vec-DNase I also significantly reduced 24-hour albuminuria, whereas ivDNase I administration did not reduce albuminuria (Figure 10A). Further evidence for prevention of glomerular injury by vec-DNase I was seen with reductions in both proportions of glomeruli affected by segmental necrosis, a reduction that was more substantial than in ivDNase I–treated mice (Figure 10, B, F, and G), and recruitment of glomerular neutrophils, macrophages, and CD4^+^ T cells (Figure 10, C–E). To further investigate the role of DNase I in clearing ecDNA, we investigated caspase-3 and RIPK3 glomerular protein expression as markers of apoptosis and necroptotic cell death. Vector delivery of rmDNase I reduced expression of these proteins in comparison with the vec-GFP group (Figure 10, F and G). Collectively, these data show that gene therapy overcomes the issue of short half-life of DNase I to provide a superior treatment option for anti-MPO GN.

### Exogenous DNase I concentrations required to inhibit NETs have no effect on neutrophil phagocytosis.

Administration of vec-DNase I can produce more than 6 months of supraphysiological amounts of DNase I (41). A concern of having a continuous supply of DNase I is that ecDNA released via infectious agents, such as CpG from bacteria, may fail to trigger pattern recognition receptors and associated pro-inflammatory cascades required to eliminate pathogens. To investigate whether using DNase I would compromise neutrophil phagocytosis, and impede host protection, we conducted both NET (Figure 10H) and pHrodo Green *S*. *aureus* bioparticle assays (Figure 10I) using thioglycolate-elicited mouse peritoneal neutrophils and PMA as a stimulant. The lowest concentration of rhDNase I to prevent NET formation was 1 μg/mL (Figure 10G) whereas all concentrations of rhDNase I between 0 and 4 μg/mL showed no significant differences between cell phagocytosis from untreated neutrophils versus neutrophils incubated with DNase I (Figure 10H), indicating that DNase I has no effect on neutrophil phagocytosis at any of the DNase I concentrations. These in vitro results suggest that DNase I can dampen inflammation while permitting neutrophil phagocytosis by healthy neutrophils.

## Discussion

It is widely accepted that NETs are a major driver of inflammation in MPO-ANCA GN through deposition of MPO, the autoantigen in the disease (17, 20, 35, 36). What is unclear is the contribution of ecDNA in perpetuating inflammation in MPO-ANCA GN. The current study strongly supports a role for ecDNA acting as a pro-inflammatory DAMP mediating injury and autoimmunity in MPO-ANCA GN. We show that ecDNA is a major feature in MPO-ANCA GN patient biopsies and that this is accompanied with a significant diminution of kidney DNase I expression. These observations were mirrored in 2 animal models of anti-MPO GN that show similar pathophysiology to human disease. We have also shown that administration of exogenous DNase I in experimental anti-MPO GN diminished NETs, decreased MPO and ecDNA deposition, and preserved kidney DNase I expression levels. Moreover, we show for the first time to our knowledge in anti-MPO GN that gene therapy using an AAV can deliver therapeutic levels of DNase I with just one dose of vector.

Our first aim in this study was to assess kidney biopsies from patients with MPO-ANCA GN for ecDNA and DNase I expression. MPO-ANCA GN biopsies had significant ecDNA deposits accompanied with a diminution in the protein expression levels of renal DNase I when compared with control patients (MCD). This indicated that DNA may play a significant injurious role in this disease and that kidney DNase I is depleted in an attempt to clear the ecDNA.

NETs are integral to the pathophysiology of ANCA vasculitis; they can be induced by ANCA (plus ANCA-independent mechanisms) and are closely associated with the deposition of extracellular MPO within MPO-ANCA GN patient biopsies (17, 36, 42). MPO bound to ecDNA has greater biological activity and is shielded from endogenous inhibitors such as ceruloplasmin (37). Together, this evidence indicates NETs are both a major driver of inflammation and source of autoantigens (17, 36, 42, 43). The capacity of exogenous DNase I to clear not only NET-deposited injurious ecDNA but also DNA deposited from all forms of cell death reported in anti-MPO GN (necroptosis, apoptosis, and necrosis) was tested in a 20-day murine model of actively induced autoimmune anti-MPO GN, which shows many of the hallmarks of human disease (20, 36, 44). MPO-ANCA GN can be characterized as both a type II antibody-mediated disease (due to the effects of ANCA) and a type IV cell-mediated hypersensitivity (due to the Th1 cell-mediated aspect of the disease) as per Gell and Coombs classification (45). We and others have shown that deposited extracellular MPO can be recognized by MPO-specific CD4^+^ T cells that in turn initiate cell-mediated glomerular injury characterized by DTH effectors, including the glomerular recruitment of M1 macrophages and the deposition of fibrin, features of GN that are prominent in human and murine anti-MPO GN (46, 47). Additional notable features of the model include generation of MPO-ANCA, patterns of glomerular injury and neutrophil infiltration (focal and segmental necrosis and leukocyte infiltration), and pathological albuminuria (48–51). Administration of DNase I to mice with established anti-MPO GN substantially reduces ecDNA, glomerular NET formation, and MPO deposition. DNase I treatment also substantially attenuated MPO-specific dermal DTH and the numbers of glomerular CD4^+^ T cells, macrophages, and neutrophils, indicative of DNase I inhibiting type IV cell-mediated disease. Importantly, exogenous DNase I treatment also preserved DNase I protein expression levels in the kidney, which is important as depletion of DNase I expression in the organ would render it even more vulnerable to pathological assault induced by uncleared NETs and DNA.

Having established that DNase I is therapeutic even after establishment of anti-MPO autoimmunity in the 20-day cell-mediated model of anti-MPO GN, we next dissected mechanisms by which DNase I reduces systemic autoimmune responses to MPO. While the mechanisms of DNase I clearance of ecDNA are well understood (34, 52, 53), the mechanisms by which this ecDNA clearance attenuates anti-MPO autoimmunity and GN is unclear. To investigate this phenomenon, we utilized a 10-day model free of complicating GN to dissect out the role of DNase I in preventing systemic autoimmunity to MPO. We revealed that continued daily treatment using exogenous DNase I after animal immunization against MPO using FCA resulted in substantial reductions in MPO-specific IFN-γ–producing cells as well as MPO-induced dermal DTH responses. However exogenous DNase I treatment failed to reduce MPO-ANCA levels. These results validate a role for DNA in the induction of anti-MPO cellular autoimmunity. The source of this DNA comes from the accompanying adjuvant, known to contain significant amounts of DNA, and from endogenous neutrophils attracted to sites of MPO immunization whereby they release NETs and DNA (54). Further supporting evidence for a role for DNA activation in MPO-ANCA GN comes from the capacity for DNA subcomponents (e.g., CpG, bacterial DNA components) alone to provide sufficient adjuvant support to allow MPO immunization to induce anti-MPO autoimmunity and GN (24, 55). Furthermore, in the 18-hour model, to dissect out the role DNase I may be playing at the time of antigen presentation, our studies show that DNase I treatment prior to MPO immunization significantly reduces the frequency of DCs in lymph nodes that drain immunization sites and that the DCs that are present show reductions in DC activation markers, including surface costimulatory molecules essential for naive anti-MPO–specific CD4^+^ T cell activation and proliferation. DNase I inhibition of DC activation and lymph node migration supports the view that DNA (in adjuvants or recruited neutrophils) is a potent inducer of MPO autoimmunity. A limitation of this experiment is that we were unable to collect sufficient cells from the draining lymph nodes from naive or OVA-immunized mice to perform reliable FACS analysis to determine the immune response to be MPO (antigen specific). The lack of lymph node expansion at this 18-hour time point in the OVA/FCA-immunized mice, however, does suggest that our results reflect an MPO-specific enlargement of the lymph nodes. This is likely due to the highly pro-inflammatory nature of MPO instigating an innate immune response coupled by early lymphocyte activation and proliferation.

DNA provides a danger signal to both macrophages and DCs, which induces them to increase their expression of MHC class II and costimulatory molecules CD40 and CD80/86 (53, 54, 56). The mechanism of DNA activation involves selective DC uptake of the autoantigen MPO by the scavenger receptor DEC-205 followed by binding to the DNA cytosolic receptor TLR9 (24, 57). Therefore, it is likely that DNase I acts by reducing local or systemic ecDNA levels, thereby circumventing activation of DC and subsequent T and B cell proliferation. MPO-pulsed DCs stimulated with the TLR9 ligand CpG (a component of DNA) stimulate Th1 responses and MPO autoimmunity and promote kidney injury in experimental anti-MPO GN (24). In contrast, mice receiving *Tlr9*^–/–^ DCs are protected from injury. Given the major ligand of TLR9 is DNA, this provides further supporting evidence that DNA is inflammatory and gives impetus to therapeutically target ecDNA (24). Furthermore, the observation that mice deficient in DNase I or DNase II develop systemic autoimmunity and glomerulonephritis provides additional support for a homeostatic/protective role for DNase I or DNase II (32, 58). Reinforcing the potential of DNase I treatment for acute renal injury are recent studies showing that exogenous DNase I is protective in a rat model of ischemia/reperfusion-induced acute kidney injury and reduces necroptosis in murine ANCA vasculitis (20, 52).

Having established the kinetics and consequence of early cell-mediated responses in the models, we then turned our attention to defining the role of ecDNA in the production and effects of MPO-ANCA, as this is highly relevant to human disease. ANCA binds to neutrophils, inducing their infiltration into glomerular capillaries, where they degranulate, produce ROS, undergo NETosis, deposit MPO extracellularly, and mediate GN. To test the robustness of our findings in the cell-mediated 20-day model of anti-MPO GN, we used a second model. We administered DNase I before administering LPS and passively transferring anti-MPO antibodies. In this 6-day model DNase I inhibited anti-MPO antibody–mediated injury by reducing the amount of ecDNA able to activate innate immune inflammatory injury through DAMP pathways.

DNase I treatment of in vitro neutrophils has shown that DNase I is associated with a decrease in ROS levels and does not directly inhibit MPO but degrades the extracellular matrix of MPO-associated DNA, which decreases the biological activity of the MPO (59). These modulating effects on neutrophils in the circulation are likely to account for the significant reduction of ANCA-induced neutrophil accumulation in glomeruli. Exogenous DNase I abrogates NET formation and ecDNA deposition and significantly reduces glomerular neutrophil accumulation, resulting in significant reductions in pathological injury (assessed by focal segmental necrosis). In this model of MPO-ANCA GN, the effects of DNase I on ANCA-mediated injury can be assessed without any confounding effects of DNase I on anti-MPO autoimmunity, as the transfer of anti-MPO antibodies means that active T and B cell autoimmunity is absent. However, as both these models study acute injury, they do not assess the effects of DNase I over the longer term development and progression of anti-MPO autoimmunity and ANCA-associated GN.

The current study demonstrates that viral vector delivery of DNase I in the 20-day model was able to diminish kidney injury (segmental necrosis and albuminuria). Vec-DNase I reduced the titers of MPO-ANCA, whereas twice daily exogenous DNase I administration did not achieve this outcome. Exogenous delivery of DNase I has also shown to be ineffective at lowering IgA and IgE levels in an animal model of IgA nephropathy (60). It is possible that the sustained and supraphysiological levels of DNase I achieved with vec-DNase I are more effective in removing the effects of ecDNA acting as a DAMP than ivDNase I, which has a shorter half-life. This demonstrates, for the first time to our knowledge, that vec-DNase I may be used as a biological therapeutic to attenuate the development or perpetuation of anti-MPO autoimmunity. As NETs are also a prominent pathophysiological feature that correlate with disease severity and depleted circulatory DNase I in patients with IgA vasculitis, it would be of great interest to see if vector delivery of DNase I has therapeutic benefit in IgA vasculitis (61).

In this study we were able to detect substantial levels of DNase I via immunohistochemistry in the mouse kidney and via Western blot in the serum. However, we were unable to successfully detect substantial levels of DNase I via Western blot in the kidney. The human protein atlas shows that the kidney has one of the highest mRNA expression levels of DNase I compared with other organs in the body, but the atlas does not show DNase I protein expression within the kidney (62, 63). This is at odds with our results and others that show strong staining for DNase I in kidney tissue from patients with MCD and that enzyme expression is depleted in patients with MPO-ANCA GN. Previous studies in lupus nephritis and membranous nephropathy have also demonstrated DNase I expression both in human and in mouse studies (26, 34, 64). In particular, patients with MPO-ANCA GN have enhanced loss of DNase I protein within the kidney compared with patients with lupus nephritis. This was also observed in our models of anti-MPO GN, where OVA-immunized mice (without autoimmunity to MPO) had substantially more DNase I protein expression than untreated mice, and exogenous DNase I partially restored this level in the kidney. It is possible that the detection of DNase I we and others have observed via immunohistochemistry is due to increased sensitivity due to preservation of DNase I via formalin fixation. DNase I is known to be sensitive to proteolytic degradation even when stored at minus 80 degrees. Furthermore, DNase I is susceptible to inactivation by G actin, which may be inhibited by fixation in formalin (65). Outside the digestive system (parotid gland, submaxillary gland, and intestine), the kidneys in human, pig, bovine, rabbit, rat, and mouse tissue have the highest levels of DNase I activity when measured by single radial enzyme diffusion (66). This points to Western blots being technically unable to detect levels of DNase I as opposed to lack of DNase I in the kidney.

One limitation of this study is the comparison of rhDNase I delivered intravenously versus murine DNase I expressed by the AAV. Murine and human DNase I share more than 80% homology, and the more than 100-fold increase in serum DNase activity supports a mechanistic explanation based on enzyme levels rather than species specificity. To better evaluate translational potential, future studies should explore the development of an AAV expressing human DNase I and assess its efficacy in a humanized model, such as a human liver chimeric mouse, to more accurately reflect clinical relevance for the treatment of anti-MPO GN. There has been considerable interest for many decades in clinically translating recombinant DNase I in multiple disease contexts. In the preantibiotic era, the enzyme was used topically to clear and sterilize infected wounds and relieve meningitis (67). Currently, it is administered in nebulized form to the airways of patients with cystic fibrosis to degrade nuclear material released from dead neutrophils, thereby reducing viscosity of respiratory secretions (68). IvDNase I has also been used in clinical trials of systemic lupus erythematosus, where it was safe and well tolerated, though ineffective in disease control possibly because of its relatively short half-life in serum (<5 hours) (69). Our study has shown gene delivery provides a solution to this issue and creates an option that is therapeutically superior to intravenous enzyme delivery. AAVs have a strong safety profile and permit long-term transgene expression in postmitotic cells (in the liver), making them ideally suited to treat chronic and remitting diseases, such as MPO-ANCA GN, which typically requires ongoing treatment that may be prolonged over years (70–72). Additionally, use of a vector that targets hepatocytes to release DNase I systemically rather than targeting the kidney directly circumvents the limitations of successful gene transfer of damaged kidney cells. As patients with MPO-ANCA GN have significantly damaged kidneys, in particular endothelial cells and podocytes, tissue pathology may be a barrier to successful gene transfer. Systemic release of DNase I via the liver hepatocytes will not only target ecDNA present in the kidney but also have further therapeutic benefit by also digesting DNA being released from cell death within the circulation. Further development of the technology to pharmacologically regulate transgene expression would offer the remarkable prospect of switching therapy “on and off” to regulate vector dosing of DNase I, thus allowing for therapy during active disease that could be discontinued during remission and reactivated in relapsing disease (73). AAV-based gene therapy has already demonstrated success in multiple clinical indications, including retinal dystrophy and spinal muscular atrophy (74, 75). Given the chronic and relapsing nature of ANCA vasculitis, a vector that enables long-term, regulatable expression of DNase I may offer a transformative therapeutic option. Our findings position DNase I gene therapy as a promising strategy to counteract ecDNA-mediated inflammation and immune dysregulation in MPO-ANCA GN.

## Methods

### Sex as a biological variable.

As MPO-ANCA GN affects both males and females equally, both sexes were included in human and mouse studies.

### Patient cohort.

Twenty-nine renal biopsies from consented patients with diagnosed MPO-ANCA GN, and 6 renal biopsies from patients with MCD, a nonproliferative form of kidney disease, with normal light microscopy, were used in this study (clinical data in Supplemental Table 1). Biopsies were collected from consented patients with ethics approval over a period from 2001 through 2016 at Monash Health, Clayton, Victoria, Australia. The biopsies from patients with MPO-ANCA GN were collected at each patient’s first presentation.

### Mice.

C57BL/6J mice were bred at Monash Medical Centre Animal Facilities, Monash University, Australia, and used at 8–10 weeks of age. *Fcgr2b*^–/–^ mice (C57BL/6J background: B6; 129S-Fcgr2b^tm1Ttk^/J) were purchased from The Jackson Laboratory and bred at Monash Medical Centre Animal Facilities, Monash University, Australia. The phenotype of *Fcgr2b*^–/–^ mice was confirmed by flow cytometry with anti-B220 and anti-CD16/CD32Ab (BD Biosciences), as previously described (75). Mice aged 8–10 weeks were used for experiments under specific pathogen–free conditions.

### Experimental design.

An established model of active anti-MPO autoimmunity was used to assess anti-MPO cell-mediated glomerular injury (48–51). WT mice (male and female) were immunized intraperitoneally with 20 μg rmMPO (76) in FCA (Sigma-Aldrich) and boosted subcutaneously with 10 μg rmMPO in FIA (Sigma-Aldrich) on day 7. Disease was initiated (triggered) by intravenous injection of 3 mg of anti-GBM globulin on day 16 (administered as 1.5 mg, 2 hours apart), 4 hours prior to treatment. Anti-MPO autoimmunity and GN were assessed 4 days later (day 20). As a control for the immunization process and the anti-GBM globulin, one group of control mice received an irrelevant antigen, OVA, instead of MPO. Treated mice with anti-MPO GN received rhDNase I 10 mg/kg (Pulmozyme, Roche) twice daily in 300 μL of normal saline intravenously, commencing 4 hours after the last anti-GBM globulin to ensure recruited neutrophils had deposited MPO to initiate disease and twice daily until termination of the experiment on day 20. Nontreated control mice were immunized with MPO and injected with anti-GBM globulin, then received vehicle injections of saline alone. For experiments using AAV8-DNase I, both the control GFP vector and rmDNase I vector were given on day 10 (intraperitoneal injection of 1 × 10^11^ vector genomes [per mouse]) and compared with intravenous delivery of recombinant DNase I (Pulmozyme, 10 mg/kg) administered twice daily (12 hours apart) from day 16 after triggering of disease with the anti-GBM antibody until termination of the experiment on day 20.

To assess MPO-ANCA–induced (humoral) glomerular injury, anti-MPO IgG generated by immunizing *Mpo^–/–^* mice as previously described (77) was passively transferred into *Fcgr2b^–/–^* mice, which are more susceptible to anti-MPO GN (78). Mice were primed with intraperitoneal LPS (0.5 μg/g) 2 hours prior to intravenous transfer of anti-MPO IgG, which activates and recruits neutrophils to glomeruli. DNase I was given twice daily intravenously on day –1 and twice daily until the end of experiments on day 6.

### EcDNA, NET, and extracellular MPO detection.

To measure ecDNA, 3 μm formalin-fixed, paraffin-embedded (FFPE) kidney sections were cleared in xylene, rehydrated in graded alcohols, immersed in antigen retrieval solution (10 mM Tris, 1 mM EDTA, pH 9.0), and boiled in a pressure cooker for 10 minutes, as described previously (38). Utilizing a pressure cooker is gentler on the tissue and allows a reduction in retrieval time, reducing heat-related damage. This is the optimal method for preserving DNA compared with protease or no antigen retrieval (79). EcDNA was assessed by using colocalization studies using DAPI (D1306, Thermo Fisher Scientific) as a marker for dsDNA (DAPI is nucleic acid specific, which preferentially binds to the AT base pairs in the mirror groove of DNA) and β-actin as a structural component of all cells to delineate glomerular and cell structures (see Supplemental Table 2 for antibody concentrations) (38). Sections were blocked in 10% chicken sera in 5% bovine serum albumin (BSA)/phosphate-buffered saline solution (PBS) for 30 minutes and stained with a rabbit anti-mouse β-actin antibody (ab8227, Abcam) at 1 μg/mL in 1% BSA/PBS, as a cell marker (and to identify glomerular regions of tissue for measurement), overnight at 4°C. Mouse IgG at the same concentration as the primary antibodies was used as isotype controls (purified from mouse serum collected in house). For secondary detection, a chicken anti-rabbit Alexa Fluor 488 antibody at 1:200 was used for 40 minutes at room temperature (A-21441, Molecular Probes, Thermo Fisher Scientific). Slides were quenched in Sudan Black B (Thermo Fisher Scientific) as outlined below and mounted with DAPI ProLong Gold mounting media (Thermo Fisher Scientific) to detect DNA. NETs and extracellular MPO were detected as published previously (18). Briefly, sections (3 μm) of FFPE tissue specimens were mounted on Superfrost Plus slides (Menzel), dewaxed, rehydrated, and pretreated with antigen retrieval solution Tris-EDTA pH 9 (10 mM Tris, 1 mM EDTA) in a pressure cooker for 10 minutes, blocked (30 minutes) in 10% chicken sera in 5% BSA/PBS (immunofluorescence), and probed with goat anti-human MPO (detects mouse MPO; AF3667 R&D Systems), rat anti-mouse CD45 (all leukocytes, to define extracellular MPO; BD Biosciences, 550539), and mouse anti-human PAD4 (ab128086, Abcam), rabbit anti-human H3Cit (ab5103, Abcam), and goat anti-human MPO (AF3667, R&D Systems) in 1% bovine serum to detect NETs, diluted in albumin/PBS for 16 hours (4°C) (see Supplemental Table 2 for antibody concentrations). Rabbit Ig, goat Ig, and mouse Ig were used as isotype controls (purified from whole serum from Sigma). Secondary detection was with either Alexa Fluor 594–conjugated chicken anti-goat IgG or Alexa Fluor 488–conjugated chicken anti-rabbit IgG, or donkey anti-mouse Alexa Fluor 647 IgG (Molecular Probes, Thermo Fisher Scientific, A-21468, A-21441, and A-31571; 1:200, 40 minutes, room temperature). To quench tissue autofluorescence, slides were incubated with Sudan Black B (0.1% in 70% ethanol, 30 minutes), washed in PBS, and coverslipped in DAPI ProLong Gold (Molecular Probes, Thermo Fisher Scientific). Fluorescent images were acquired using a Nikon C1 confocal laser scanning head attached to Nikon Ti-E inverted microscope (Coherent Scientific); 405, 488, 561 and 647 nm lasers were used to specifically excite DAPI, Alexa Fluor 488, Alexa Fluor 594, and Alexa Fluor 647. Single-plane 512 × 512 × 12 bit images were captured in a line-sequential manner (4-line averaging) using a 20×, 40×, or 60× objective.

### Assessment of ecDNA and extracellular MPO.

To assess ecDNA in human renal biopsies and murine kidneys, our previously published method using supervised machine learning for semiquantification of ecDNA was used. In brief this method uses an open source, trainable ImageJ plugin for Weka segmentation. This computational method uses classifiers to train the model to recognize ecDNA outside a cell. This model is then saved and applied to all other images. The “analyze particle” feature in ImageJ was used and circularity set to include nuclear staining (DAPI), and the nuclear stain was thresholded, marked, and excluded from analysis using the watershed function in ImageJ, leaving only the extranuclear stain (area DAPI-positive outside the nucleus) to be measured (ImageJ program open access NIH with Weka segmentation plug-in) (38). The results were then expressed in arbitrary units per glomerular cross section. Extracellular MPO was measured by a previously published method using a macro in ImageJ analysis software (36). Intracellular (leukocyte-associated) MPO was defined as being associated with CD45 (CD45^+^MPO^+^ cells). Extracellular MPO was defined and measured as MPO^+^CD45^–^ staining. The macro evaluated both the area and intensity and expressed the results as AU.

### Statistics.

The Shapiro-Wilk test for normality revealed all data were nonparametric. Therefore, results are expressed as either the median and interquartile range or median and standard deviation. Kruskal-Wallis 1-way ANOVA was used for comparisons between 3 groups, and a Dunn post hoc test was performed for multiple comparisons of groups. Mann-Whitney *U* 1-tailed test was used for nonparametric data and paired *t* test for comparison in ex vivo coculture experiments. The OVA-immunized mice (reference control) were excluded from multiple comparisons tests. All data were analyzed with GraphPad Version 10.4 (Prism; GraphPad Software Inc.). Differences were considered statistically significant if *P* < 0.05; data *P* > 0.05 were not indicated.

### Study approval.

Study approval for use of patient kidney biopsy material and use of clinical data was given by the Monash Health Ethics Committee (application number 08216B). Written informed consent was received prior to participation. Study approval for the animal work within the study was given by Monash University’s animal ethics committee, approved application numbers MMCB2019/35, MMCB2014/23, and MMCB2017/43.

### Data availability.

Supporting data values can be found in the accompanying Supporting Data Values XLS file. Images used for analysis are available upon request from the corresponding author. Supplemental Methods are available online with this article (for DNase I staining, RIPK3 and caspase-3 staining, flow cytometry of lymph nodes, and MPO-specific ELISPOTs).

## Author contributions

KMO, GJL, PYG, ARK, and IEA conceptualized the study. KMO, ACL, PYG, VO, JD, MAA, MM, LL, TAG, KEL, DKYC, and GJL conducted the experiments. KMO, PYG, JD, MAA, MM, LL, TAG, KEL, and GJL analyzed the data. KMO wrote the first draft and made the figures. KMO, GJL, PYG, IEA, and ARK reviewed and edited the paper. KMO, GJL, and PYG acquired the funding. All authors approved the final version of the manuscript.

## Supplementary Material

Supplemental data

Unedited blot and gel images

Supporting data values

## Version 1 07/09/2025

In-Press Preview

## Version 2 08/08/2025

Electronic publication
