# Supplementary material for: Gene therapy enhances deoxyribonuclease I treatment in antimyeloperoxidase glomerulonephritis
Source: JCI Insight. 2025 Jul 8;10(15):e188951. doi: 10.1172/jci.insight.188951 (PMC12333947; doi:10.1172/jci.insight.188951)
Supplement: Supplemental data [file jciinsight-10-188951-s127.pdf]

Supplementary Tables:

Table 1. Clinical Data from patients with ANCA associated Vasculitis

Patient Characteristics	**MPO-ANCA-associated vasculitis**	**MCD**
Patient number	29	6
Age	63.5 (10)	44 (±15)
Sex F/M	15/14	4/2
Number of glomeruli	18	19
Laboratory values		
MPO-ANCA titre (U/ml)	149.58 (±23)	n/a
eGFR (ml/min/1.73 m^2)	34.77 (±8)	100.1 (±6)
ESR (mm/h)	70 (±8)	n/a
CRP (mmol/l)	66.4 (±25)	n/a
Urinary red blood cells (cells/HPF)	552 (±15)	n/a

Abbreviations: ANCA, anti-neutrophil cytoplasmic antibody; CRP, C reactive protein; eGFR, estimated glomerular filtration rate; ESR, erythrocyte sedimentation rate; MCD, minimal change disease MPO, myeloperoxidase. Values reported as Median and standard deviation.

Table 2. Antibodies

Antibody	Specificity	Company	Dilution
mouse anti human β-actin	β-actin	Abcam, ab8227	1μg/mL
Goat anti - human/mouse MPO	myeloperoxidase	R&D Systems, AF3667	10μg/mL
Rat anti mouse CD45	CD45, leukocytes	BD Biosciences 550539	6ug/mL
Mouse anti human/mouse PAD4	PAD4	Abcam, ab128086	10μg/mL
Rabbit anti human/mouse H3Cit	Citrullinate histones	Abcam, ab5103	20μg/mL
Mouse anti human/mouse DNase I	DNase I	Santa Cruz	1/100
Rabbit anti mouse active Caspase 3	Active Caspase 3	Australian Biosearch 9664	5μg/mL
Rabbit anti human/mouse RIPK3	RIPK3	Sapphire Bioscience	5μg/mL
Rat anti mouse CD4 (GK1.5)	CD4 T cells	ATCC, cell line	20μg/mL
Rat anti mouse CD68 (FA/11)	CD68 macrophages	Cell line from Dr Gordon L. Loch Cambridge	10μg/mL
Rat anti mouse Gr1 (RB6-8C5)	Gr1, neutrophils	Bio X Cell	5μg/mL
Rat ant mouse Ly6g	Ly6g, neutrophils	Bio X Cell	5μg/mL

Abbreviations: ATCC, American Type Culture Collection; CD, cluster differentiation; BD, Becton Dickinson; PAD4, Peptidyl arginine deiminase ;R&D Systems, Research and Development.

A

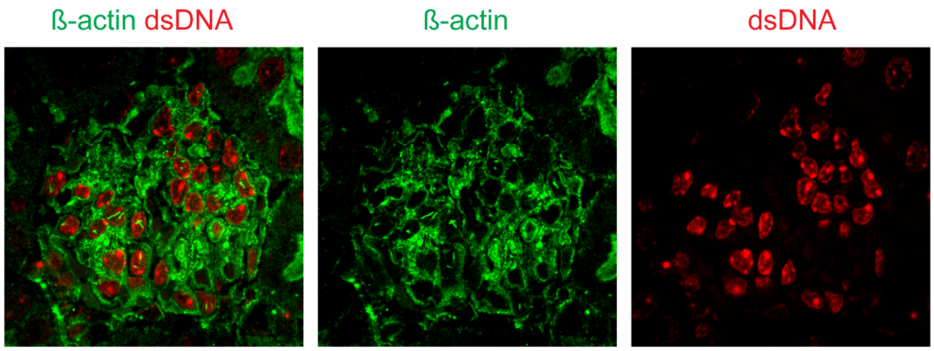

Supplementary Figure 1. Extracellular DNA is minimally observed in naïve C57/Bl6 mice.

(A) Kidney sections from a normal mouse stained for DNA (red) and β-actin (green). Original magnification 200x

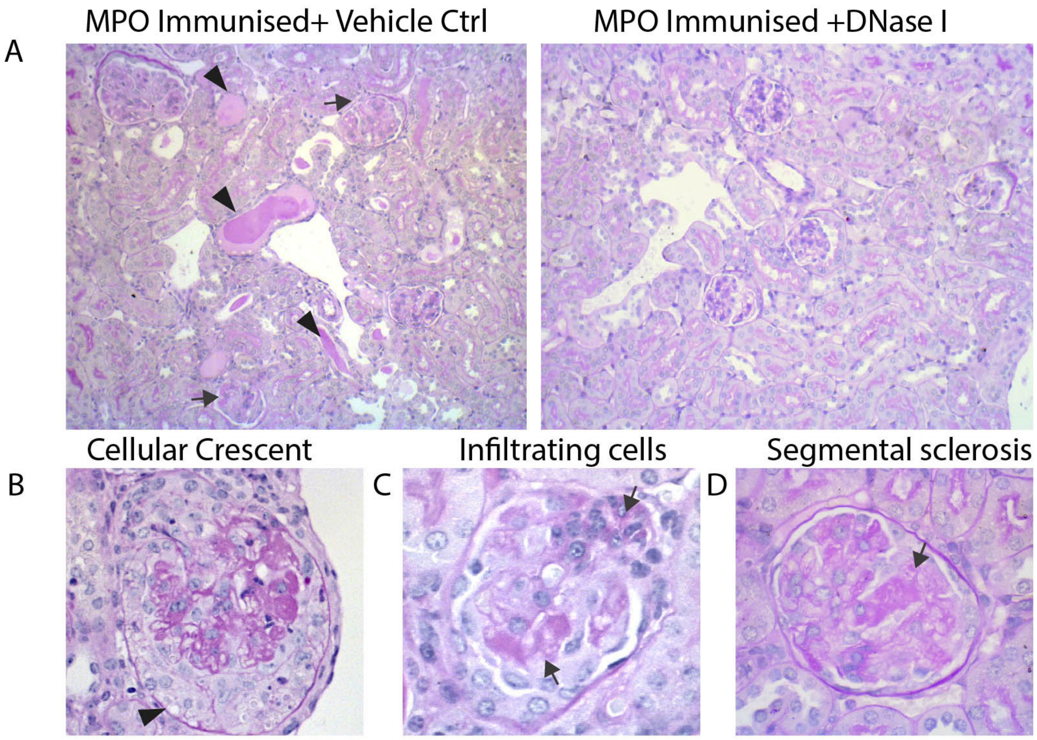

Supplementary Figure 2. Low powered images demonstrating extent of histological damage between the vehicle control group and treatment groups and examples of histological injury observed. (**A**) Low powered Image of the tubulointerstitium showing multiple tubular casts (arrow heads) and damaged glomeruli (arrows) compared to DNase I treated kidney with no tubular casts and minimal histological changes to glomeruli. (**B**) Example of cellular crescent, arrow pointing to crescent within Bowman's capsule(**C**) Infiltrating cells into Bowman's space and area of segmental necrosis, arrows (**D**) examples of segmental sclerosis/necrosis. Abbreviations: MPO, myeloperoxidase, DNase I, deoxyribonuclease I, Ctrl. Control. Original magnification for (A-B) 200x, original magnification for (B-D) 400x.

Supplementary Figure 3

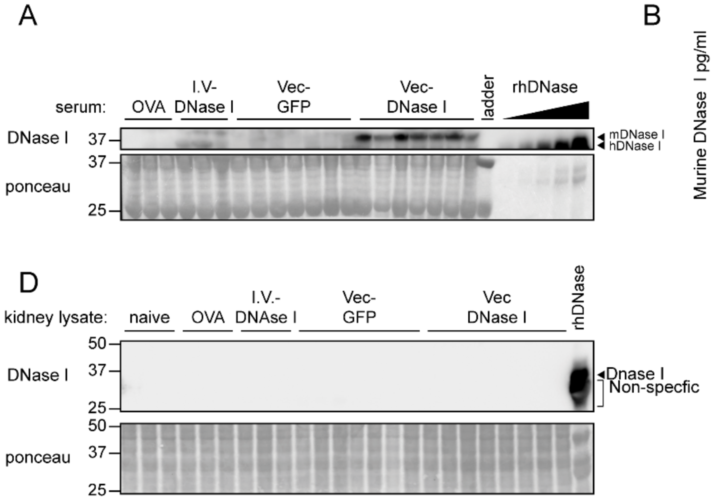

Supplementary Figure 3. Western blots for DNase I in mouse serum and kidney lysate
(A) Western blot showing serum levels of human DNase I in the i.v DNase I treated group and mouse murine DNase I in the Ve-DNase I group, **(B-C)** Quantification of human and murine DNase I in serum and **(D)** western blot for DNase I on kidney lysates demonstrate no detectable level of DNase I Abbreviations: rhDNase I, intravenous recombinant human deoxyribonuclease I, Ctrl. mDnase I, Murine DNase I; Vec, Vector; Ova, ovalbumin; GFP, green fluorescent protein.*** P<0.001

Supplementary Methods

Adeno-associated viral vector production, DNase I activity assay and quantification of liver vector copy number.

AAV vectors encoding GFP or and DNase I packaged into AAV serotype 8 (AAV8) capsids were purified and titrated as previously (41). Vectors were injected into the intraperitoneal cavity at 1 x 10^{11} vector genomes (vg) per animal. At the end of the 20-day anti MPO-GN model livers were removed and examined for vector copy number as previously published (41). Vector copy number was determined using Platinum Taq DNA polymerase (Invitro-gen) according to the manufacturer's instructions, using primers specific for the WPRE region on the vector and normalized to GAPDH. DNase I activity from plasma samples were determined using radial enzyme diffusion assays according to our previously published protocol (41). The halo diameter around each well of digested DNA was measured and compared to DNase I standards.

Immunoblotting

Kidney tissue lysates were prepared as previously described (Tye et al. Life Science Alliance 2024). Briefly, snap frozen kidney tissue was ground by mortar and pestle and lysed at 167mg/1ml RIPA buffer (150 mM NaCl, 50 mM Tris [pH 7.4], 1 mM deoxycholate, 1% [vol/vol] Triton X) containing complete protease inhibitor cocktail (Roche) and 1mM sodium pervanadate for 1hr on a rotating wheel at 4oC. Insoluble content was pelleted by centrifugation at 17000 x g for 10 min, and the clarified lysate collected. Lysate protein concentration was quantified by Pierce BCA Protein assay kit (ThermoFisher) and 30μg equivalent of lysate was prepared in 1× NuPAGE LDS (Thermo Fisher Scientific) + 5% β-mercaptoethanol. Serum samples were prepared by adding 2μl to 1× NuPAGE LDS (Thermo Fisher Scientific) + 5% β-mercaptoethanol. Recombinant human DNase I was prepared as a positive control; kidney blots were ran with 10pg equivalent DNase I and serum blots were ran

with 10pg, 5pg, 2.5pg, 1.25pg and 0.625pg equivalent DNase I all in 1× NuPAGE LDS (Thermo Fisher Scientific) + 5% β-mercaptoethanol.

Prepared lysate, serum and recombinant DNase I samples were boiled at 95oC for 10min prior to SDS-PAGE. Samples were separated using 4–12% Bis-Tris gradient gels (NW04125/27BOX; Invitrogen), then transferred to nitrocellulose membranes (Millipore). Ponceau staining was used to demonstrate protein loading. Membranes were blocked in TBS + 0.1% Tween-20 (TBS-T) + 5% skim milk powder for 30min, then incubated with 1:1000 mouse anti-human DNase I (Santa Cruz) in TBS-T + 0.04% sodium azide overnight at 4oC. Blots were washed in TBS-T and incubated with polyclonal goat anti-mouse immunoglobulin HRP conjugate (DAKO) for 1 hr at RT, before imaging with a Bio-Rad ChemiDoc MP. Images were analysed and processed with Bio-Rad ImageLab software. Serum DNase I concentrations were determined by plotting sample densitometric values against a standard curve derived from the serial dilutions of control recombinant DNase I.

Assessment of renal human and mouse DNase I, Caspase 3 and RIPK3

DNase I immunohistochemistry was performed on 3 μm thick, formalin-fixed, paraffin- embedded human renal biopsies from MPO-ANCA-associated vasculitis patients and MCD patients, and murine models of T cell mediated anti-MPO GN and GN induced by passive transfer of anti-MPO antibodies. Sections were cleared in Histosol, rehydrated in graded alcohols and then blocked with 10% Horse serum in 5% BSA/PBS for 30 minutes at RT and incubated with either a mouse anti-human DNase I antibody (1/100, Santa Cruz, overnight at 4 degrees), rabbit anti human/mouse receptor-interacting serine/threonine-protein kinase 3 (RIPK3, 5μg/mL) or rabbit anti mouse active caspase 3 (Australian Biosearch, 5μg/mL). Rabbit Ig and mouse Ig were used as isotype controls (purified from whole serum in house). Sections were washed, and endogenous peroxidase blocked with 1% Hydrogen Peroxide (H_2O_2) in methanol for 20 minutes, followed by blocking with avidin

and biotin using a commercially available blocking kit per manufacturer's instructions (Vector Laboratories). Secondary antibody detection was performed with a horse anti-mouse biotinylated antibody (for DNase I) at 1:60 (Vector Laboratories) or Swine anti rabbit biotinylated antibody 1/500 (DAKO) for 40 minutes at RT, and detected with an avidin biotin complex conjugated to HRP (ABC-HRP) for 40 minutes (Vector Laboratories), then with 3,3′-diaminobenzidine (DAB brown, SIGMA), dehydrated, cleared in Histosol and mounted in Vector Shield (Vector Laboratories) permanent mounting media. Five low power (20x objective lens) fields of view of the tubulointerstitium were analyzed and graded 1-3 according to the amount of stain present within tubulointerstitial epithelial cells in human renal biopsies, and 10 interstitial fields of view were analyzed in mice.

Assessment of renal injury and leukocyte infiltration

Histologic assessment of renal injury was performed on 3 μm thick, FFPE fixed, periodic acid-Schiff-stained kidney sections. At least 50 consecutive glomeruli/mouse were examined and results expressed as percentage of abnormal glomeruli exhibiting either; crescent formation, segmental necrosis, sclerosis or infiltrating immune cells and expressed per glomerular cross-section (gcs). Glomerular CD4+ T cells, macrophages, and neutrophils were assessed by an immunoperoxidase-staining technique on 6 μm thick, periodate lysine paraformaldehyde (PLP) fixed, OCT frozen kidney sections. The primary antibodies used were GK1.5 for CD4+ T cells (anti-mouse CD4+; American Type Culture Collection), FA/11 for macrophages (anti-mouse CD68 from Dr. Gordon L. Koch, Cambridge, England), and RB6–8CS for neutrophils (anti- GR-1; Bio X Cell). Isotype controls Rat IgG2A and IgG2B were used as isotype controls as the concentration as the primary antibodies (BD Pharmigen). At least 30 glomeruli were assessed and results expressed as cells/gcs (c/gcs).

Systemic Immune Responses to MPO

ELISA was used to detect circulating serum anti-MPO IgG titers using 100µl/well, 1µg/ml murine MPO and horseradish peroxidase conjugated sheep anti-mouse IgG (1:1000; Amersham Biosciences) ELISPOT plates were incubated overnight with IFN-γ and IL-17A coating antibody in sterile PBS.(BD Biosciences, Mouse IFN-γ ELISPOT kit and Mouse IL-17A ELISPOT kit) .Draining LN cells were seeded at $5x10^5$ cells/well re-stimulated with 10 µg/ml of rmMPO for 18 hours. IL17A and IFNγ secondary biotinylated antibody from the ELISPOT kit were used to detect IFN-γ and IL-17A producing cells followed by a streptavidin HRP detection antibody which was detected by 3-Amino-9-ethylcarbazole (AEC). AEC positive spots were enumerated with an automated ELISPOT reader system (TECAN). To assess MPO-specific dermal delayed type hypersensitivity (DTH), mice were challenged by intradermal injection of 10 µg murine MPO in 30µl saline in the right hind footpad (the contralateral footpad received saline). DTH was quantified 24 hours later by measuring the difference between footpad thicknesses (Δmm) using a micrometer, the user was not blinded as the automatic micrometer removed bias.

Flow cytometry and intracellular staining

DCs were identified as $CD11c^{hi}$ cells on isolated draining LN cells by flow cytometry. LN cells were stained for 30 minutes at 4˚C with the following directly conjugated antibodies: hamster anti-mouse CD11c PE (HL3), hamster anti-mouse CD11c PerCP/Cy5.5 (N418, BioLegend), rat anti-mouse CD40 FITC (3/32, BD Biosciences), rat anti-mouse MHC- II APC/Cy7 (M5/114.15.2, BioLegend), rat anti-mouse ICOS Ligand PE (HK5.3, Biolegend), mouse anti-mouse OX40L PE (8F4, BioLegend), hamster anti-mouse CD80 FITC (16-10A1, eBioscience), rat anti-mouse CD86 APC/Cy7 (GL-1, BioLegend). CD4 (GK1.5, BD), CD69 (H1.2F3, BD). For analysis of MPO specific Treg responses ex vivo, 5 x 10^5 LN cells were cultured for 72 hours with 10µg/ml MPO. CTV, as per manufacturer's instructions was added at 0 hours and cells stained using anti-Foxp3 (FJK-16s, EBioscience) and Foxp3 fixperm kit (Ebioscience). Cells were analyzed on the Beckman Coulter Navios platform and data analyzed using FlowJo software (TreeStar).

NET phagocytosis Assays

For the in vitro NET assay and zymosan phagocytosis assay *n*=5 C57BL/6J male mice were injected with aged 4% thioglycolate intraperitoneally (500µl). Neutrophils (2x10^5) were plated out in 24 well plates for immunostaining, then pre-incubated with DNase I at varying concentrations from 0-4ug/ml DNase I for 30 minutes prior to adding PMA (40µg/ml, SIGMA) for 3 hours at 37°C to induce NET formation in serum free RPMI (SIGMA) media [to avoid blocking of NET production with albumin. Cells were fixed in paraformaldehyde (2%) supplemented with periodate and lysine overnight at 4°C. Cells were washed, permeabilized, blocked in 10% chicken sera and then stained for markers of NETs, goat anti human/mouse myeloperoxidase (anti-MPO, AF3667, R&D Systems), rabbit anti human/mouse citrullinated histone 3 (H3Cit, Ab5103, Abcam), and mouse anti human/mouse peptidyl arginine deiminase 4 (PAD4; Ab128086, Abcam), and detected with secondary chicken anti-rabbit AF488 (Thermofisher), chicken and mouse AF647 (Thermofisher), and chicken anti- goat AF594 (Thermofisher) and mounted in DAPI ProLong Gold (Molecular Probes, Thermo Fisher Scientific). NETs were visualized by confocal fluorescence microscopy with a Nikon Ti- E inverted microscope (Nikon Instruments). Then, 405-, 488-, 561-, and 647- nm lasers were used to specifically excite DAPI, Alexa Fluor 488, Alexa Fluor 594, and Alexa Fluor 647.

For the phagocytosis assays neutrophils obtained from thioglycolate peritoneal exudate as described above from *n*=7 C57BL6 mice were plated out at 5x10^5 into a 96 well plate and allowed to settle for 30 minutes at 37°C prior to the addition of 0-4µg/ml DNase I. 30 minutes after the addition of DNase I PMA at 40µg/ml was added for 3 hours with pHrodo™ Green *S. aureus* Bioparticles™ as per manufacturer's instructions (pHrodo™ Green *S. aureus* Bioparticles™ Kit, P35382, ThermoFisher Scientific), visualized and read at an absorbance plate reader at OD 509/533 nm (Tecan). As all treated cells were above the reading of the untreated cells (eg above 100%) results were displayed as OD reading rather than percentage phagocytosis.

Real-Time PCR for gene expression

Kidneys were stored at minus 80 degrees until time of RNA extraction. RNA extraction was performed via a standard protocol using trizol, a High capacity cDNA reverse Transcription Kit (Applied Biosystems, Foster City, CA) was used to generate cDNA. Taqman Universal PCR Master Mix and Taqman Gene expressions assays were performed as per the manufactures instructions and normalized to the house keeping gene *18S* (Applied Biosystems, Gene specificities for (*Ccl2, Cxcl1, Cxcl2, Ifng, Il1b Il6, Tnf* and *DnaseI*) and read on the 7900HT Fast Real-Time PCR System (Applied Biosystems).
