# Supplementary figures and images for: Gene therapy enhances deoxyribonuclease I treatment in antimyeloperoxidase glomerulonephritis
Source: JCI Insight. 2025 Jul 8;10(15):e188951. doi: 10.1172/jci.insight.188951 (PMC12333947; doi:10.1172/jci.insight.188951)

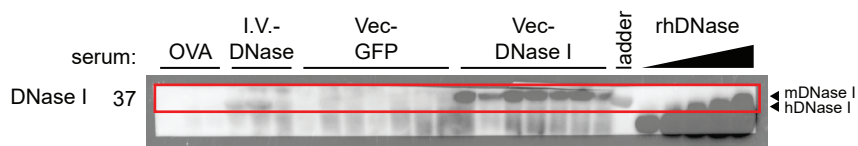

ponceau (serum):

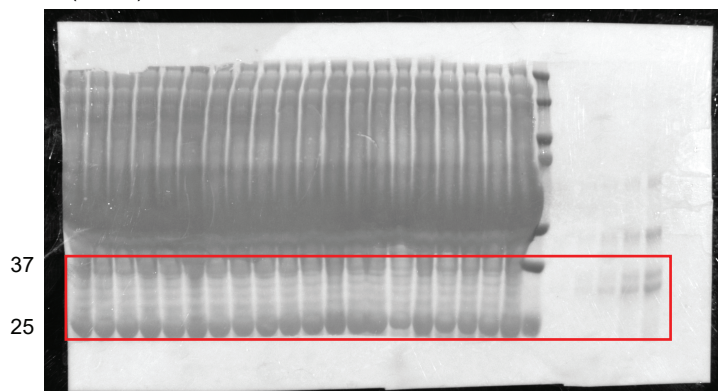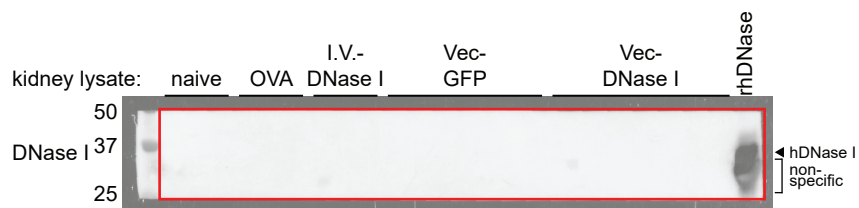

ponceau (kidney lysate):

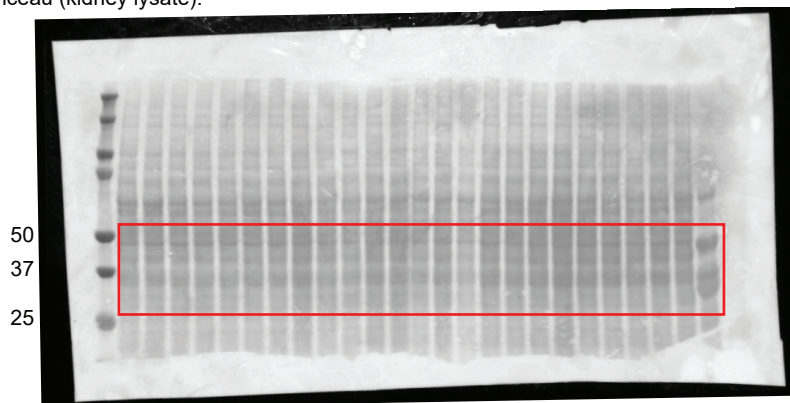

Supplement: Unedited blot and gel images [file jciinsight-10-188951-s128.pdf]
